# Ultra‐Microporous Fe‐MOF with Prolonged NO Delivery in Biological Media for Therapeutic Application

**DOI:** 10.1002/smll.202405649

**Published:** 2024-09-12

**Authors:** Rosana V. Pinto, Chen‐Chen Cao, Pengbo Lyu, Iurii Dovgaliuk, William Shepard, Eric Rivière, Cheng‐Yong Su, Guillaume Maurin, Fernando Antunes, João Pires, Vânia André, Carlos Henriques, Antoine Tissot, Moisés L. Pinto, Christian Serre

**Affiliations:** ^1^ CERENA Departamento de Engenharia Química Instituto Superior Técnico Universidade de Lisboa Lisboa 1049‐001 Portugal; ^2^ CQE ‐Centro de Química Estrutural Institute of Molecular Sciences Departamento de Química e Bioquímica Faculdade de Ciências Universidade de Lisboa Lisboa 1749‐016 Portugal; ^3^ Institut des Matériaux Poreux de Paris Ecole Normale Supérieure ESPCI Paris CNRS PSL University Paris 75005 France; ^4^ MOE Laboratory of Bioinorganic and Synthetic Chemistry Lehn Institute of Functional Materials School of Chemistry Sun Yat‐Sen University Guangzhou 510275 China; ^5^ ICGM Univ. Montpellier CNRS ENSCM Montpellier 34293 France; ^6^ Hunan Provincial Key Laboratory of Thin Film Materials and Devices School of Materials Science and Engineering Xiangtan University Xiangtan 411105 China; ^7^ Synchrotron SOLEIL L'Orme des Merisiers Départementale 128 Saint‐Aubin 91190 France; ^8^ Institut de Chimie Moléculaire et des Matériaux d'Orsay Université Paris‐Saclay CNRS ICMMO Orsay Cedex 91405 France; ^9^ State Key Laboratory of Applied Organic Chemistry Lanzhou University Lanzhou 730000 China; ^10^ CQE – Centro de Química Institute of Molecular Sciences Instituto Superior Técnico Universidade de Lisboa Av. Rovisco Pais Lisboa 1049‐001 Portugal

**Keywords:** biological stability, iron(III)‐MOF, nitric oxide donors, open metal sites, phosphonate

## Abstract

Nitric oxide (NO), a key element in the regulation of essential biological mechanisms, presents huge potential as therapeutic agent in the treatment and prevention of chronic diseases. Metal‐organic frameworks (MOFs) with open metal sites are promising carriers for NO therapies but delivering it over an extended period in biological media remains a great challenge due to i) a fast degradation of the material in body fluids and/or ii) a rapid replacement of NO by water molecules onto the Lewis acid sites. Here, a new ultra‐narrow pores Fe bisphosphonate MOF, denoted MIP‐210(Fe) or Fe(H_2_O)(Hmbpa) (H_4_mbpa = p‐xylenediphosphonic acid) is described that adsorbs NO due to an unprecedented sorption mechanism: coordination of NO through the Fe(III) sites is unusually preferred, replacing bound water, and creating a stable interaction with the free H_2_O and P‐OH groups delimiting the ultra‐narrow pores. This, associated with the high chemical stability of the MOF in body fluids, enables an unprecedented slow replacement of NO by water molecules in biological media, achieving an extraordinarily extended NO delivery time over at least 70 h, exceeding by far the NO kinetics release reported with others porous materials, paving the way for the development of safe and successful gas therapies.

## Graphical abstract

New ultra‐narrow pores iron bisphosphonate MOF (MIP‐210) with outstanding properties for an unprecedent controlled release of a potential gaseous therapeutic agent ‐ nitric oxide (NO). The unique adsorption mechanism, along with the MOF's high chemical stability and narrow pores, leads to an extended NO delivery time exceeding 70 h in biological media.

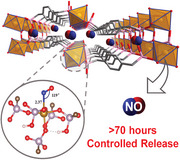

## 1 Introduction

**Figure 1:**
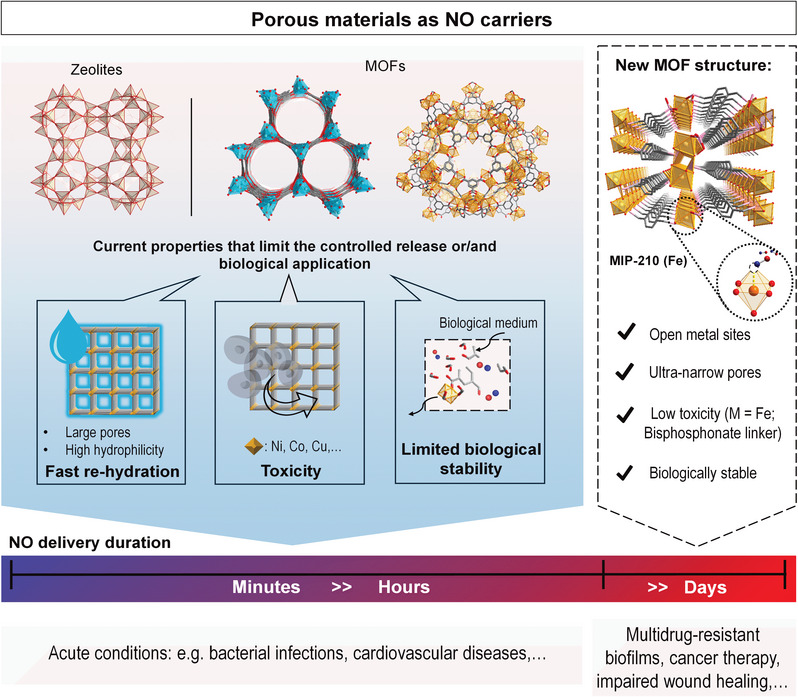
Examples of different NO‐releasing porous materials and respective limitations that inspired the development of MIP‐210(Fe). The combination of unique properties leads to a more controlled and extended NO delivery from MIP‐210(Fe) compared to the existent NO‐releasing porous solids expands its use to several therapeutic applications.

The exogenous administration of nitric oxide (NO) at controlled amounts (pmol to µmol levels) has been proved to be of therapeutic interest for many applications linked to acute and chronic diseases, i.e., cancer, cardiovascular diseases, bacterial infection, and wound healing.^[^ ^1^, ^2^, ^3^ ^]^ Recent works^[^ ^4^, ^5^, ^6^, ^7^ ^]^ unveiled that porous materials, in particular metal‐organic frameworks (MOFs), are among the most suitable and versatile scaffolds to carry and release NO since their tunable composition allows the development of stable and biocompatible frameworks that can i) be modified post‐synthetically to carry covalent NO donor moieties (i.e., diazeniumdiolates), ii) be used as catalysts to induce the release of NO directly from endogenous and supplemented S‐nitrosothiol donors^[^ ^6^, ^8^, ^9^ ^]^ or iii) carry the NO gas via its adsorption into their pores through grafting onto the open metal sites.^[^ ^5^, ^10^, ^11^ ^]^ This latest strategy has prompted the development of this work, since with adsorption we can reach highly efficient packing of NO and local delivery of pure NO without the cytotoxic leakage of metal ions/ decomposition products (**Figure** **1**).

The NO adsorption mechanism in MOFs is typically governed by either i) physisorption corresponding to non‐bonded host/guest interactions or ii) chemisorption associated with chemical‐coordination through open metal sites (OMS) present in the pore walls.^[^ ^4^, ^12^ ^]^ This later NO‐coordination mode not only allows tuning of the NO payload by changing the metal or the concentration of accessible OMS, but also ensures a more stable binding of NO.

The release of the adsorbed NO in biological media is mainly triggered by free water or phosphate molecules that replace NO on the OMS owing to their stronger affinity, which, along with the fast degradation that features the vast majority of MOFs, restricts the release lifetimes to a maximum of 2 h as shown previously by the robust Ti tetracarboxylate MOF denoted MIP‐177(Ti).^[^ ^5^ ^]^ With this release window, MOFs can only be used in therapies that require short periods of supplementation.

For the long‐term NO therapies, the only carriers releasing NO from several hours to weeks are composite polymers with molecular NO donors.^[^ ^13^ ^]^ These chemical donors become therapeutically relevant only when incorporated into hybrid systems, as many of them have short half‐lives and limited stability that may pose challenges for dosage control. Additionally, some examples may produce reactive intermediates like peroxynitrite or leach carcinogenic N‐nitrosamines, underscoring the importance of careful consideration in their utilization.^[^ ^14^, ^15^, ^16^ ^]^

To address the demanding challenge of long‐term NO delivery in body fluids, we developed a new microporous Fe bisphosphonate, namely MIP‐210(Fe) (MIP stands for the materials of the Institute of Porous Materials of Paris), comprising H_4_mbpa (or p‐xylene diphosphonic acid) and iron(III) that accommodates narrow pores and iron(III) oxoclusters with accessible OMS. Iron(III) MOFs are of particular interest for biomedical applications owing to their high biocompatibility.^[^ ^17^ ^]^ Few of them, e.g. MIL‐100 and MIL‐127, have already proven to be attractive NO carriers although hampered due to their limited stability associated with a fast NO release once in body fluids thus restricting mainly their utilization to wound healing or anti‐thrombogenic applications with a release occurring in the gas phase.^[5,^ ^10^ ^]^ Long et al.^[^ ^18^ ^]^ explored NO sorption in F_2_(dobdc) and demonstrated a stronger binding on Fe sites compared to Co_2_(dobdc) and Ni_2_(dobdc),^[^ ^11^ ^]^ resulting from an electron transfer from the Fe^II^ sites to NO to form stable Fe^III^ ‐NO^−^ adducts.

This motivated us to further explore the chemistry of metal(III/IV) phosphonate MOFs as they are known to be i) significantly more chemically stable compared to their metal carboxylate counterparts due to the higher complexing strength of phosphonates and ii) exhibit much dense architectures including narrow pores. If a few water‐stable microporous metal(III/IV) bis, tris, or tetra phosphonates MOFs have previously been reported,^[^ ^19^, ^20^, ^21^ ^]^ to our knowledge an iron(III) phosphonate MOF with accessible OMS to bind guest molecules such as NO has not been discovered so far, due either to the presence of Cl counter anions^[^ ^22^ ^]^ and/or the dense framework using a short organic spacer.^[^ ^23^ ^]^ One exception lies in the MIL‐91(Ti, Al) solids, constructed from a piperazine bisphosphonic acid under hydrothermal conditions, that are robust narrow pores microporous MOFs,^[^ ^19^ ^]^ however bearing chains of corner‐sharing metal octahedra, thus without any OMS. These MOFs were also shown to be hydrothermally stable.^[^ ^24^ ^]^ This inspired us to reinvestigate the chemistry of Fe(III) bisphosphonates under hydrothermal conditions but selecting another related bisphosphonic acid ligand, denoted H_4_mbpa (H_4_mbpa = p‐xylenediphosphonic acid), constructed from an aromatic organic spacer, expecting to obtain microporous narrow pores architectures, eventually containing OMS (Figure 1).

## 2 Results and Discussion

### 2.1 Synthesis, Structure Determination and Stability

MIP‐210(Fe) was obtained by hydrothermal synthesis (see details in Section 1), leading to parallelepiped‐shaped crystals with a uniform size distribution (Figure S1, Supporting Information). Infrared spectroscopy indicates that the solid contains partially deprotonated H_4_mbpa linkers, while EDX analysis evidences that the Fe:ligand ratio is 1:1 (Figure S3 and Table S1, Supporting Information). The structure of MIP‐210(Fe) was solved from single crystal data at 100 K using a microfocused beamline at the Soleil Synchrotron. It results in the formula Fe^III^(H_2_O)[O_3_P‐CH_2_‐C_6_H_4_‐CH_2_‐PO_3_H]·H_2_O (as‐synthesized form).

The MOF crystallizes in a monoclinic space group *P*2_1_/*n* (S.G. n°13) with unit‐cell parameters (at 100 K) of: *a*  =  5.1440(3) Å, *b*  = 10.7106(7) Å, *c* = 21.0654(9) Å and β = 93.222(4)°. The bond distances of the MIP‐210(Fe), ranging between 1.99–2.11 Å (Table S3, Supporting Information), are consistent with the usual distances between phosphonate and the Fe sites being in the oxidation state +III.^[^ ^25^ ^]^ This was confirmed by performing magnetic measurements on MIP‐210(Fe). The data (Figure S6, Supporting Information) evidenced a X_m_T product of 3.83 cm^3^ K mol^−1^ at 300 K that progressively decreased upon cooling down to 0.27 cm^3^ K mol^−1^ at 2 K, which is perfectly consistent with a compound built with weakly antiferromagnetically coupled Fe(III) cations.

**Figure 2:**
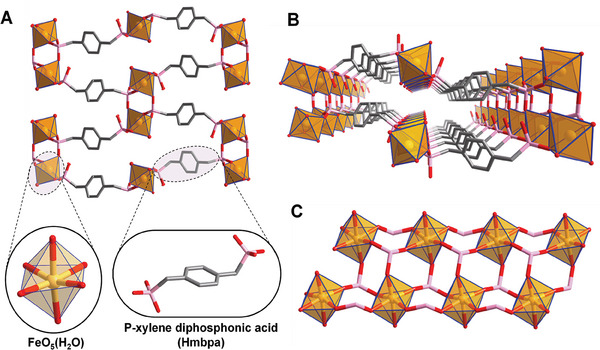
Crystal structure of MIP‐210(Fe). A) General view of MIP‐210(Fe) at the a axis, highlighting the linker (shown in its fully deprotonation form although in the structure one of the phosphonate groups is only partially deprotonated (R‐HPO_3_^−^) coordinated to two adjacent Fe(III) cations) and FeO_5_(H_2_O) polyhedra (H_2_O is omitted for clarity). The bonded water can either be removed and create an OMS or can be replaced by NO. B) Representation of one channel along the [1 0 0] emphasizing its highly confined environment. C) Double FeO_5_(H_2_O) chains in MIP‐210(Fe) viewed at the [0 0 1] (H_2_O is omitted for clarity). Color code: FeO_5_(H_2_O), dark yellow polyhedra; O, red; C, grey; P, pink. Hydrogen atoms on the MOF framework (and on the linker representation) and non‐coordinated guest molecules have been omitted for clarity.

The asymmetric unit contains one p‐xylenediphosphonic acid linker and one Fe(III) cation (**Figure** **2**). Each linker possesses one fully deprotonated phosphonate group (R‐PO_3_^2−^) coordinated to three adjacent Fe(III) cations and one partially deprotonated phosphonate group (R‐HPO_3_^−^) coordinated to two adjacent Fe(III) cations (Figures 2A and S7, Supporting Information for more clear illustration). Noteworthy, if Fe(III) cations are surrounded by five oxygen atoms from five different phosphonate linkers, it is completed by one oxygen atom from a coordinated water molecule, leading to a pseudo‐octahedral geometry. These coordination modes give rise to double chains of Fe(III) along the *a* axis that are linked through the phosphonate linker (Figure 2C).

In addition, the structure, although not adsorbing N_2_ at 77 K, presents ultra‐microporous corrugated channels along the *a* axis that contain one terminal water molecule per Fe(III) cation in its pristine form (Figure 2A,B). The simulated Powder X‐ray Diffraction (PXRD) pattern of the crystal structure matches well with the corresponding experimental data, which confirms the purity of the synthesized powder (Figures S4 and S5, Table S2, Supporting Information). In addition, variable temperature PXRD indicated that the overall framework holds over 250 °C, in agreement with thermogravimetric analysis (Figures S8 and S9, Supporting Information) while it undergoes a reversible tiny structural change ≈150 °C due to the removal of the water molecules (Figures S3,S5, S8, and S10, Supporting Information). MIP‐210 also proved to be remarkably stable in water at different pH (Figure S11, Supporting Information), in phosphate‐buffered saline (PBS)(pH 7.4) and Tris buffer (pH 8.5) up to 10 days (Figures S12 and S13, Supporting Information) and in several biological media at 37 °C (Figures S14–S17, Tables S4–S6, Supporting Information), confirming the required stability to be considered as a delivery NO platform (detailed discussion in Section S2.3, Supporting Information).

### 2.2 NO Adsorption Mechanism

**Figure 3:**
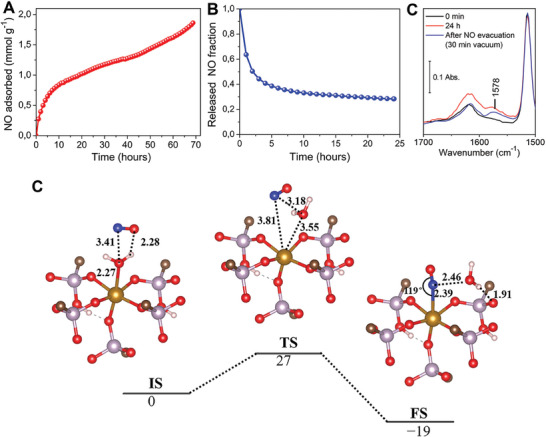
Experimental NO adsorption studies at 25 °C on MIP‐210(Fe) and computational understanding of the NO adsorption mechanism. A) Gravimetric NO adsorption profile for 80 kPa NO; B) NO desorption profile under high vacuum and C) IR spectra for the sample previously activated at 120 °C for 12 h under vacuum, followed by 24 h of NO exposure at room temperature (1.3 kPa) and its evacuation (30 min of vacuum). D) periodic DFT‐calculated potential energy profile for the adsorption of NO in MIP‐210(Fe) with Fe initially coordinated by water molecule using climbing image nudged elastic band (CI‐NEB)^[^ ^30^ ^]^ method. Color codes: carbon (dark brown), hydrogen (white), oxygen (red), phosphorus (purple), nitrogen (blue), and iron (light brown). The distances and energies are in Å and kJ mol^−1^, respectively. For the sake of clarity, only one inorganic node of the periodic structure is shown.

NO adsorption on MIP‐210 at 80 kPa was carried out after activating the solid at different temperatures to maximize the NO loading (detailed discussion in Section S3, Supporting Information). The MOF once activated at 120 °C under vacuum, allowing the removal of only physiosorbed water molecules, led to a substantial adsorption capacity of 1.86 mmol · g^−1^ after 3 days (**Figure** **3**A). At higher activation temperatures, there is a removal of both free and coordinated water molecules that led to a slight contraction of the pores leading to lower adsorption capacities (Figure S21, Supporting Information). The experimental amount of NO loaded is lower than the theoretical number of OMS of this MOF (≈3 mmol · g^−1^), suggesting an incomplete loading even after 72 h (mass still not constant, Figure 3A) probably due to the presence of very narrow pores that slows down the diffusion of NO toward the open metal sites. The amount adsorbed lies however in the range of the reported MOFs studied for NO application (0.8–7mmol · g^−1^),^[^ ^11^, ^26^, ^27^ ^]^ with a slightly lower capacity compared to other MOFs showing Fe(III) OMS, i.e., Fe‐MIL‐100, Fe‐MIL‐127 and Fe‐MIL‐88‐A with 4.5, 2.2, and 2.5 mmol g^−1^ NO adsorption capacities, respectively,^[^ ^10^, ^28^ ^]^ and with comparable theoretical OMS (≈3.6 mmol g^−1^ (MIL‐100); ≈2.7 mmol g^−1^ (MIL‐127); ≈3.8 mmol g^−1^ (MIL‐88A)).

The desorption curve under vacuum in Figure 3B revealed an initial NO release in the first 2 h of ≈50% of the total adsorption capacity and a very slow delivery after that period. After 24 h under primary vacuum, ca. 0.56 mmol · g^−1^, i.e., 30% of the adsorbed NO, remains in the porous material, indicating a strong NO adsorption, which is beneficial for higher control of the therapeutic release and for safer storage of the loaded material.

To shed light on the NO adsorption mechanism in MIP‐210, in situ FTIR studies were first conducted. IR spectra (Figure 3C) on the activated sample at 120 °C exhibit the appearance of a NO adsorption band at 1578 cm^−1^ after 24 h of exposure to NO, which increases in intensity with time (Figure S20, Supporting Information) in line with the slow NO adsorption profile (Figure 3A). Noteworthy, no band is observed at ca. 1900 cm^−1^ typically assigned to physisorbed NO, i.e., weak interactions between NO and the MOF framework.^[^ ^28^ ^]^ Thus, we can assume that only chemisorption occurs, which is the most favorable adsorption mechanism for NO therapeutic delivery applications. After 30 min under high vacuum, despite losing some intensity, the band formed at 1578 cm^−1^ persisted, confirming the strong stability of the adsorbed NO species, in agreement with the gravimetric desorption studies (Figure 3B) that showed a significant NO retention in the solid. Of note, the IR adsorption band observed in MIP‐210(Fe) upon NO adsorption does not match with the typical band assigned to the NO coordinated on Fe sites (ca. 1800 cm^−1^) observed in the previously studied Fe‐based MOFs (MIL‐100, MIL‐127, MIL‐88′s) after NO adsorption.^[^ ^10^, ^28^ ^]^ According to Hadjiivanov,^[^ ^29^ ^]^ bands formed between 1650 and 1480 cm^−1^ are associated with the formation of nitrate (NO_3_^−^) compounds. However, NO_3_^−^ is not likely to form here because NO cannot form NO_3_^−^ just by reacting with H_2_O solely. To gain more insights into the impact of NO adsorption on the MIP‐210(Fe) crystalline structure, in situ‐PXRD analyses were conducted. After NO adsorption, the main Bragg peaks were shifted, in comparison with those of the activated phase (i.e., without physisorbed water in the pores), becoming similar to the as‐synthesized material, in agreement with a filling of the pores (Figure S23 and Table S2, Supporting Information).

In complement to this experimental characterization, periodic spin‐polarized density functional theory (DFT) calculations were further performed to gain atomistic insight into the NO adsorption behavior in MIP‐210(Fe). First, we identified that the most stable magnetic configuration of MIP‐210(Fe) is an intra‐chain anti‐ferromagnetic (AFM) state, as shown in Figure S24 and Table S7 (Supporting Information). Climbing image nudged elastic band (CI‐NEB)^[^ ^30^ ^]^ method within the Transition State Tools for VASP (VTST)^[^ ^31^ ^]^ module (see Section S3.1, Supporting Information for more details) was further applied to explore the NO adsorption in the MOF encompassing Fe(III) sites coordinated to H_2_O to be in line with the activation condition used experimentally before NO adsorption measurement. As shown in Figure 3D), H_2_O coordinates to Fe with an O‐end configuration (Fe─O bond length 2.27 Å) and NO molecule weakly interacts with the H_2_O molecule via O(NO)···H(H_2_O) hydrogen bond (2.28 Å) (initial state IS). Then, the water molecule is released from Fe, and Fe–O distance enlarges to 3.55 Å while the NO molecule starts to get close to Fe with Fe–N distance of 3.81 Å in the transition state (TS). In the final state (FS), NO molecule binds to Fe with a Fe─N bond length of 2.39 Å and a Fe–N–O angle of 119°. According to a previous study on N–O stretching frequency of NO molecule within 6‐coordinate iron(II) nitrosyl complexes, there is a linear scaling relationship between the Fe–N–O angle and the *v* (NO).^[^ ^32^ ^]^ While a vibration at 1900 cm^−1^ would correspond to a Fe–N–O angle of 180°, the *v* (NO) corresponding to a Fe–N–O angle of 119° should be extrapolated between 1550 and 1600 cm^−1^, in line with the NO adsorption band at 1578 cm^−1^ observed in the experimental IR spectrum in Figure 3C. Notably, this NO coordination to Fe(III) is accompanied by rather strong interactions between NO and H_2_O (N(NO)‐O(H_2_O) distance of 2.46 Å) as well as between H_2_O and the MOF pore wall (H(H_2_O)‐O(MOF) distance of 1.91 Å) making this adsorption mode highly stable, therefore anticipating that a slow release kinetics in water media should be obtained. Further, the overall reaction is exothermic by 19 kJ mol^−1^, and the activation barrier is only 27 kJ mol^−1^, which indicates that the H_2_O exchange by NO can easily proceed under mild conditions leading to preferential binding of NO toward Fe(III). The calculations presented in Figure 3D) were performed considering as a starting point the crystal structure based on the close proximity between the PXRD patterns of the NO loaded sample and the as‐synthesized hydrated MOF. A similar trend was obtained considering the crystal structure solved for the material filled with water molecules (Figures S25 and S26, Supporting Information), the reaction mechanism being associated with an even lower activation energy barrier (11 kJ mol^−1^) and being more exothermic (40 kJ mol^−1^).

### 2.3 NO Therapeutic Release Application

**Figure 4:**
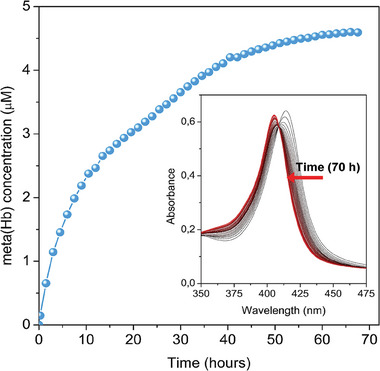
NO release profile from MIP‐210 in oxyhemoglobin solution at 25 °C using the oxyhemoglobin quantification assay. Inset depicts the changes over 70 h in the main peak of the oxyhemoglobin‐containing solution spectrum, triggered by NO‐mediated conversion to methemoglobin. The concentration of meta(Hb) quantified is considered stoichiometric to the concentration of NO released.^[^ ^36^ ^]^

The effectiveness of a carrier material for NO release is mainly determined by its ability to release NO at a controlled rate since the NO biological action strongly depends on the available dose (lower or higher values may cause adverse effects) over a certain period. This feature highly depends on how NO is coordinated to the framework and on the accessibility of water present in the biological media toward OMS. As such, quantification of NO released from MIP‐210(Fe) was carried out in oxyhemoglobin solution using the oxyhemoglobin assay (**Figure** **4**), exhibiting an exceptionally slow and gradual delivery of NO over at least 70 h, surpassing by far the performance observed before with the best MOFs and porous inorganic materials.^[^ ^26^ ^]^ In Figure S22 (Supporting Information), this release profile can be compared with the profiles from representative NO‐releasing MOFs (MIL‐100(Fe)^[^ ^10^ ^]^ and MIP‐177(Ti)^[^ ^5^ ^]^) and a LTA zeolite (4A)^[^ ^33^ ^]^ by using the same quantification method. This method is very suitable for detecting NO being released at low concentrations (as we see for MIP‐210), offers real‐time monitoring in biological media, and is highly specific to NO by specifically measuring the reaction of NO with oxyhemoglobin to form methemoglobin. Of note, the stabilization of the conversion in methemoglobin observed at the end of 70 h may indicate a complete release or full consumption of the oxyhemoglobin present in the buffer medium. Overall, this method is not able to quantify the complete release profile and hence the total amount of NO released (for that, complementary methods should be considered), but it is very sensitive and selective during the presence of oxyhemoglobin and thus very convenient to show the initial release slope. Particularly for MIP‐210, it demonstrates an initial very slow NO release rate, as confirmed by the slow conversion to methemoglobin (Figure 4 inset). Such a slow release is attributed to i) the unusual NO adsorption mechanism described above that leads to strong interactions between NO, the Fe metal sites and the water molecules inside the narrow pockets of the structure, ii) the very narrow channels of the MOF and iii) the high stability of MIP‐210 in several biological media, making the displacement of the coordinated NO by H_2_O during the desorption process very slow.

The previously reported NO‐releasing porous materials were limited to a delivery from minutes to a few hours due to either fast rehydration and/or degradation of the host solid.^[^ ^5^ ^]^ Typically MIP‐177(Ti),^[^ ^5^ ^]^ a robust microporous Ti_12_O_15_ oxocluster‐based tetracarboxylate MOF, features a set of suitable properties for such application, including high storage capacity, biocompatibility, and stability in biological media, as well as accessible OMS, but albeit the unusual formation of nitrites species, due to its large pore size (1,1 nm), water diffusion is too fast, with subsequent hydrolysis of nitrites and consequently release of NO that does not last more than 2 h once soaked in simulated body fluid (Figure S22, Supporting Information). On the other hand, zeolites and titanosilicates, which are highly stable and biocompatible narrow pores materials, present a strong affinity to water that leads to an initial burst of NO release on a time scale that never exceeds several minutes.^[^ ^33^, ^34^ ^]^ The longest release in simulated body fluid obtained by this class of materials was 4 h by a modified ETS‐4 with copper incorporated in the structure.^[^ ^35^ ^]^

In addition to its singular release lifetime, MIP‐210(Fe) guarantees therefore a much slower release from the beginning of contact with the biological media, preventing the initial burst of release observed in many reported NO‐delivery strategies.^[^ ^13^ ^]^ This gradual and long release is important to provide a continuous modulation of the biological processes and to help mitigate the potential for cytotoxicity and tissue damage by minimizing fluctuations in NO levels maintaining NO concentrations within a safe and physiological range. Such a NO delivery profile is comparable with those obtained by some NO‐functionalized polymers and some nanoparticle carriers that already demonstrate therapeutic potential in several pre‐clinical studies, encouraging the use of this MOF in numerous biomedical applications.^[^ ^13^, ^37^, ^38^ ^]^ For instance, a poly(ε‐caprolactone)/chitosan (PCL/CS) dressing with grafted glycosylated diazeniumdiolates demonstrated a similar sustained NO release profile of at least 3 days (catalyzed by galactosidade).^[^ ^39^ ^]^ This dressing was then applied in the treatment of full‐thickness skin wounds in mice, demonstrating accelerated wound healing when compared to both control and PCL/CS‐only groups.

Remarkably, the NO released by different concentrations of MIP‐210(Fe) did not induce any additional noxious/toxic effect on HeLa (human cervical carcinoma cells) and HUVEC cells (primary human umbilical vein cells, responsible for the formation of blood vessels) when compared to the same concentration of material without NO (Figures S28 and S29, Supporting Information). This highlights the importance of controlled release that ensures the safe use of this donor. In contrast, uncontrolled release, particularly at high concentrations caused by a burst release (typical of many compounds), might lead to off‐target effects such as cytotoxicity and tissue damage, thereby limiting their therapeutic potential.^[^ ^15^, ^16^, ^40^ ^]^

In addition, if our MOF is stable over days of contact with PBS pH 7.4 (see Supporting Information for details), iron‐based MOFs are expected to be fully biodegradable and poorly toxic even after administration of high doses.^[^ ^41^ ^]^ After clear evidence of the biocompatibility of MIP‐210(Fe) under different cell lines (detailed discussion in Section S4, Supporting Information), first demonstrations of the therapeutic potential of this new NO donor were conducted in vitro by performing endothelial cell migration and tube formation assays. These cellular responses are two pivotal steps in the angiogenesis process, which in turn is critical for many physiologic and pathologic processes such as wound healing and tissue remodeling.^[^ ^42^ ^]^ MIP‐210 is of particular interest for this application, offering several advantages over the standard daily wound dressing replacement approach, namely: 1) sustained therapeutic effect: NO may work more effectively when released gradually over time rather than in a single dose; 2) reduced frequency of dressing changes; 3) stable wound environment by providing a consistent level of NO or moisture, which is crucial for the creation of a permanent antimicrobial surface and for the optimal tissue remodeling; 4) cost‐effectiveness and 5) enhanced wound management: certain wounds, such as chronic ulcers or large surgical incisions, may benefit from extended exposure of NO provided by MIP‐210.

**Figure 5:**
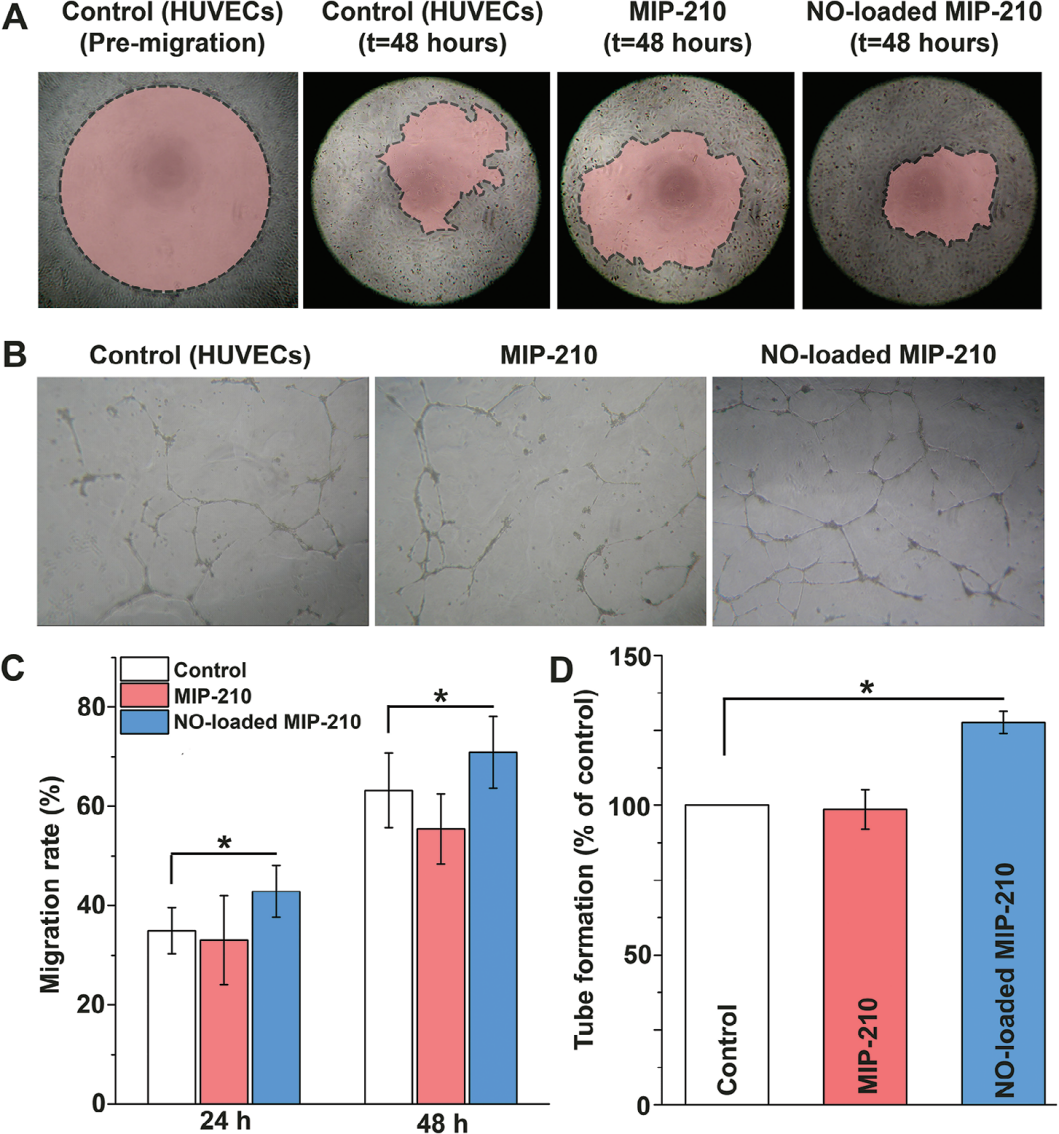
Effects of NO released by MIP‐210(Fe) on HUVEC cells migration and on Geltrex tube‐forming assay. A) Representative images of the Oris cell migration assay in the pre‐migration stage and after allowing cells to migrate in the presence (or not) of the MIP‐210(Fe) (unloaded and loaded with NO) at the concentration of 11.75 µg mL^−1^. Colored areas represent the migration zone that is still not occupied by cells. B) Example images of HUVEC tube formation after being treated with (or without) NO‐releasing MIP‐210(Fe) (11.75 µg mL^−1^) after 18 h. C) HUVEC cells migration data after 24 and 48 h. D) Quantification of tube formation (measured by the number of branches) after 18 h. Graph values are expressed as mean ± SD (n ≥ 3) and statistical differences were performed using unpaired t‐test student (^*^ *P* < 0.05).

HUVEC cells treated with NO‐loaded MIP‐210(Fe) (11.75 µg mL^−1^ – concentration selected according to the discussion in Supporting Information) showed a fast migration (ca. 8%, *p* < 0.05) after 24 and 48 h compared to the untreated cells and to the cells treated with pristine MIP‐210(Fe) (**Figure** **5**A–C and S30, Supporting Information). Endothelial tube formation was also potentiated by the released NO from MIP‐210(Fe) with a significantly richer network (ca. 25%) in comparison with the cell control and the pristine MIP‐210(Fe) groups (Figure 5B–D). Both cellular responses demonstrate that the released NO is the main contributor to the enhanced migration and tube formation, giving us confidence in the potential angiogenic capacity of the NO‐loaded MIP‐210(Fe) at the cellular level. We recognize the importance of further validating these therapeutic benefits with more in vitro tests and in an in vivo setting but to the best of our knowledge, this is the first evidence of the use of a porous solid as a carrier for NO therapeutic pro‐angiogenic targeting delivery. As MOFs are relatively new in gas therapy applications, their biological assessment is still very scarce. Still, a previous report used a different cell line (HeLa cells) to demonstrate a similar improvement in the cell migration (+ 8.4 ± 1.4% comparing with the control) induced by the NO released by MIP‐177.^[^ ^5^ ^]^ Moreover, a study presented by Zhang et al.^[^ ^7^ ^]^ showed a NO‐releasing system composed of a N‐diazeniumdiolate modified copper‐based MOF (HKUST‐1) incorporated in polymeric nanofibers capable of significantly inducing HUVEC proliferation, migration, and tube formation due to the gradual NO release (1.74 nmol L^−1^ h^−1^) over more than 14 days.^[^ ^43^ ^]^ The incorporation in polymer support might indeed constitute a possible follow‐up direction for MIP‐210(Fe), in which poorly toxic composites based on this ultra‐stable MOF might enhance the NO release performance, assuring even longer periods, reduced side effects, and simpler handling in a clinical context. This includes, for instance, the possibility to be formulated with thin films, hydrogels, and coatings, allowing for versatile administration routes such as local implantation, topical application, or systemic injection. This formulation approach will also afford the opportunity to subsequently adjust the release kinetics,^[^ ^44^, ^45^, ^46^ ^]^ enabling the customization of therapeutic effects based on specific biological or clinical needs. Future studies will aim to explore various formulations and different applications, designed to possess specific properties and functionalities aligned with distinct therapeutic needs.

One possible example is to explore MIP‐210 as antithrombogenic and antibacterial agents in coatings in medical devices to improve their performance when in contact with biological fluids or tissues.^[^ ^45^, ^47^ ^]^ Another highly relevant application is wound healing promoter for chronical wounds in patients with impaired tissue regeneration (e.g., diabetes).^[^ ^13^ ^]^ In this case, one can imagine enhancing the antibacterial efficacy of our system by formulating MIP‐210 with another MOF that features a short‐release profile with high NO levels (crucial to eradicate the bacterial burden at the surface). This dual NO release profile could potentially be more effective against chronic wound infections. This type of formulation with different MOFs that feature different release profiles was already investigated by Ettlinger et al..^[^ ^48^ ^]^ Since NO has a central role in cancer and inflammatory diseases, therapies based in NO delivery are under development and the release from MIP‐210(Fe) platforms may also be a safe option to explore.^[^ ^49^ ^]^

It is worth noting that formulations of molecular donors with polymers have been extensively explored for several decades, which is reflected in formulations in more advanced stages of clinical validation, as for instance the first N‐diazeinumdiolate‐based NO‐releasing therapeutic FDA approved in January of this year (Zelsuvmi), underscoring the promising prospects for MIP‐210. In any case, the NO‐releasing performance itself has convincingly proven an unprecedented release profile with no apparent toxicity for cells and a preliminary conception of its therapeutic potential.

## Conflict of Interest

The authors declare no conflict of interest.

## Supporting information



Supporting Information

## Data Availability

The data that support the findings of this study are available from the corresponding author upon reasonable request.
