# Supplementary material for: Ultra‐Microporous Fe‐MOF with Prolonged NO Delivery in Biological Media for Therapeutic Application
Source: Small. 2024 Sep 12;20(48):2405649. doi: 10.1002/smll.202405649 (PMC11600697; doi:10.1002/smll.202405649)
Supplement: Supplementary file 1 — Supporting Information [file SMLL-20-2405649-s001.pdf]

NANO · MICRO
small

Supporting Information

for *Small*, DOI 10.1002/smll.202405649

Ultra-Microporous Fe-MOF with Prolonged NO Delivery in Biological Media for Therapeutic Application

Rosana V. Pinto, Chen-Chen Cao, Pengbo Lyu, Iurii Dovgaliuk, William Shepard, Eric Rivière, Cheng-Yong Su, Guillaume Maurin, Fernando Antunes, João Pires, Vânia André, Carlos Henriques, Antoine Tissot, Moisés L. Pinto* and Christian Serre**

Supplementary Information

Ultra-Microporous Fe-MOF with Prolonged NO Delivery in Biological Media for Therapeutic Application

Rosana V. Pinto# [a,d], Chen-Chen Cao# [b,c], Pengbo Lyu [e,f], Iurii Dovgaliuk,[a], William Shepard [g], Eric Rivière [h], Cheng-Yong Su [c,i], Guillaume Maurin [e], Fernando Antunes[d], João Pires[d], Vânia André[j], Carlos Henriques[j], Antoine Tissot* [b], Moisés L.Pinto* [a] and Christian Serre* [b]

# These authors contributed equally to this work.

[a] Dr. R. V. Pinto, Prof. M. L. Pinto
CERENA. Departamento de Engenharia Química, Instituto Superior Técnico, Universidade de Lisboa, 1049-001 Lisboa, Portugal
E-mail: moises.pinto@tecnico.ulisboa.pt
[b] Dr. C. Cao, Dr. I. Dovgaliuk, Dr. A. Tissot, Dr. C. Serre
Institut des Matériaux Poreux de Paris, Ecole Normale Supérieure, ESPCI Paris, CNRS, PSL University, 75005 Paris, France
E-mail: antoine.tissot@ens.psl.eu / christian.serre@ens.psl.eu
[c] Dr. C. Cao, Prof. C. Su
MOE Laboratory of Bioinorganic and Synthetic Chemistry, Lehn Institute of Functional Materials, School of Chemistry, Sun Yat-Sen University, Guangzhou 510275, China
[d] Dr. R. V. Pinto, Prof. F. Antunes, Prof. J. Pires
CQE -Centro de Química Estrutural, Institute of Molecular Sciences, Departamento de Química e Bioquímica, Faculdade de Ciências, Universidade de Lisboa. Lisboa, Portugal.
[e] Dr. P. Lyu, Prof. G. Maurin
ICGM, Univ. Montpellier, CNRS, ENSCM, Montpellier, France
[f] Dr. P. Lyu
Hunan Provincial Key Laboratory of Thin Film Materials and Devices, School of Materials Science and Engineering, Xiangtan University, Xiangtan 411105, China
[g] Dr. W. Shepard
Synchrotron SOLEIL, Gif-Sur-Yvette, France
[h] Dr. E. Rivière, Institut de Chimie Moléculaire et des Matériaux d'Orsay, Université Paris-Saclay, CNRS, ICMMO, 91405 Orsay Cedex, France
[i] Prof. C. Su
State Key Laboratory of Applied Organic Chemistry, Lanzhou University, Lanzhou 730000, China
[j] V. André, Prof. C. Henriques
CQE - Centro de Química Estrutural – Instituto Superior Técnico, Universidade de Lisboa. Av. Rovisco Pais, 1049-001 Lisboa, Portugal

Contents

Contents 2
Section 1 - Experimental Section/Methods 3
Section 2 - MOF characterization 9
2.1. Structure Determination 12
2.2. MOF thermal and chemical stability 15
2.2.1 Stability tests in biological conditions 19
2.3. Detailed discussion on MIL-210(Fe) stability 23
Section 3 - NO adsorption/release detail 24
3.1. Molecular simulation 28
Section 4 - Cell culture and *in vitro* studies 31
4.1 Detailed discussion on MIP-210(Fe) biocompatibility and *in vitro* studies 34
Bibliography 36

Section 1 - Experimental Section/Methods

Chemicals and Cells: All chemicals were purchased commercially and used as received without further purification. P-xylene diphosphonic acid (H_4mbpa), 99%, Sikemia. Ferric chloride hexahydrate, 98%, Fisher. Ethanol absolute, ≥99%, Acros. MilliQ water, Millipore system, Sodium hydrosulphite (≥82% (RT)) and haemoglobin human lyophilized powder, Sigma-Aldrich, Nitric oxide gas (99.99%), Air Liquide.

HeLa cells were obtained from American Type Culture Collection (Manassas, VA, USA), HEKn cells from Thermo Fisher Scientific and HUVEC from Live Technologies. RPMI-1640 without L-glutamine, Corning Inc.; penicillin-streptomycin, L-glutamine, foetal bovine serum (FBS), trypsin (2.5%, without phenol red), Epilife medium with 60 µM calcium, human keratinocyte growth supplement kit, Medium 200 PRF, low serum growth supplement (LSGS), trypsin-EDTA (0.25% without phenol red), trypsin neutralizer solution and Geltrex™ LDEV-Free Reduced Growth Factor Basement Membrane Matrix were all purchased from Thermo Fisher Scientific.

Synthesis of H_4mbpa ligand: The synthesis was carried out by Sikemia using the Arbuzov reaction starting with the corresponding dibromo-p-xylene following the protocol described elsewhere[1].

Synthesis of MIP-210(Fe): $FeCl_3 \cdot 6H_2O$ (150 mg, 0.555 mmol) and H_4mbpa (111 mg, 0.417 mmol) were mixed with 5 mL of water in a 50 mL glass vial, sonicated for 15 minutes and then placed in an oven at 120 °C for 20 hours. The solid was washed with water and ethanol and the product was collected by centrifugation and dried under ambient atmosphere, leading to 120 mg of a bright yellow solid, which corresponds to a yield of 90 % based on ligand due to the excess of metal salt. This synthesis can also be performed in Teflon reactor in the same conditions to obtain uniform MIP-210(Fe) crystals (Figure S1).

This synthesis can be scaled up five times without any change of sample's quality. Besides, the same product can be obtained using different iron salt/ligand molar ratios (from 0.5 to 1.5) using similar conditions. It can also be obtained using any water volume between 5 to 10 mL and by adding formic acid or acetic acid as modulator.

Synthesis of MIP-210(Fe)-HT: A phase change happened when MIP-210(Fe) was heated at 120 °C under vacuum for 5 hours, leading to MIP-210(Fe)-HT.

Characterization of MIP-210(Fe): Powder X-ray diffraction (PXRD) data were recorded on a high-throughput Bruker D8 Advance diffractometer working on transmission mode and equipped with a focusing Göbel mirror producing CuKα radiation (λ=1.5418 Å) and a LynxEye detector. SEM-EDX characterization was performed with a FEI Magellan 400 scanning electron microscope. TGA data were collected on Mettler Toledo TGA/DSC 2, STAR System apparatus with a heating rate of 5 ºC / min. under oxygen or nitrogen flow. Crystal structure was obtained by single-crystal synchrotron diffraction data collected with micro-focused X-rays on the Proxima2A beamline (Synchrotron SOLEIL) using a suitable single crystal. Samples were analysed by FT-IR spectroscopy by using a Nicolet 6700 FTIR spectrometer equipped with a DTGS detector. Temperature dependent PXRD data were recorded with sample closely packed in quartz capillary on a PANalytical EMPYREAN diffractometer with CuKα radiation (λ = 1.5418 Å) and equipped with an HTK-1200N (Anton Parr) high-temperature chamber and a GaliPIX3D detector.

Stability tests in biological conditions: Structural integrity of MIP-210(Fe) after being in contact with different supplemented cell culture media (RPMI-1640, Medium 200 and EpiLife – media used for HeLa, HUVEC and HEKn cells subculture, respectively) was evaluated under biological conditions (37 ºC, humidified atmosphere with 5 % CO_2). The concentration of material tested (450 µg/mL) was the same used to perform toxicity tests with these three cell lines. After 72 hours (maximum time of the accomplished cellular assays), iron concentration in each medium was quantified (after MOF removal by centrifugation) by inductive coupled plasma atomic emission spectroscopy (ICP-AES).

NO adsorption/desorption isotherms: MIP-210(Fe) (ca. 50 mg) was first outgassed for 12 hours at 120 ºC in vacuum, cooled up to 25 ºC and the adsorption isotherm was recorded after 80 kPa of NO being introduced to the system (equipment is set to only work under atmospheric pressure for safety reasons). NO adsorption and desorption isotherms were carried out using a gravimetric adsorption system (CI Electronics, Disbal microbalance), with 0.1 µg of precision, associated with a high vacuum system composed by turbomolecular and diaphragm pumps (Pfeiffer Vacuum). Over 72 hours, the sample weight was recorded every minute with both temperature and pressure controlled by using a water bath (Grant, GD120) and using a

capacitance transducer (Pfeiffer Vacuum, CMR 262). Desorption of NO gas adsorbed was conducted in a similar manner by gradually evacuating the NO and maintaining the vacuum for 24 hours.

In situ IR characterization: IR measurements were performed to follow the NO adsorption process using an IR *operando* setup comprising a reactor-cell associated to a high vacuum system (Pfeiffer). Sample was shaped in self-supported disks (16 mm diameter, 25 mg) and pretreated at different temperatures under vacuum (Figure S21). 1.3 kPa NO was then introduced in the cell and left in contact with the solid for 24 hours at room temperature. IR spectra were recorded periodically (128 scans, 4 cm^{-1} resolution) using a Nexus-Thermo Nicolet apparatus. After that period, NO was removed under secondary vacuum.

High resolution PXRD characterization: PXRD patterns of the pristine MIP-210(Fe), activated MIP-210(Fe) and NO-loaded MIP-210(Fe) were collected using Empyrean Malvern Panalytical diffractometer with CuKα1 radiation (λ = 1.54056 Å) at room temperature in the sealed capillaries. Briefly, pristine MIP-210(Fe) was inserted directly in a capillary while for the other conditions, MIP-210(Fe) was inserted in a capillary and activated under vacuum at 120 ºC for 12 hours. The capillary corresponding to the activated MOF was sealed and the material was kept inside the capillary under vacuum for XRD analysis. For the NO-loaded MIP-210(Fe) case, the NO was inserted inside the capillary after MOF activation, and it was sealed with the NO inside for XRD analysis.

Magnetic measurements: Magnetic measurements were carried out by using a MPMS 5 Quantum Design SQUID Magnetometer. $\chi_M T$ vs T measurement was recorded under an applied field of 1 kOe. Susceptibility data were corrected for diamagnetic contributions of the sample holder and of the sample powder as deduced by using Pascal's constant tables.

NO release kinetics: After pretreatment at 120 ºC for 12 hours, MIP-210(Fe) was exposed to 80 kPa NO in a glass vacuum cell for 72 hours. After the loading period, the non-adsorbed NO was evacuated, and the sample kept in helium atmosphere until application. NO released from MIP-210(Fe) in liquid phase was quantified using the oxyhemoglobin assay. Previously described by Feelisch *et al.*[2], this spectrophotometric technique allow the NO quantification in aerobic oxyhemoglobin solution by monitorization of the stoichiometric conversion by NO of oxyhemoglobin to methemoglobin. Briefly, MIP-210(Fe) was ground together with

poly(tetrafluoroethylene) (PTFE) (25% wt), the mixture was pressed into pellets and loaded with NO in the same conditions as for the powder. Oxyhemoglobin solution was prepared by dissolving 20 mg of lyophilized human hemoglobin in 1 mL buffer solution and sodium dithionite was added for complete reduction of oxyhemoglobin. The solution was purified and desalted using a Sephadex G-25 column. For NO release quantification, the loaded MOF was placed in a quartz cuvette with 3 mL of oxyhemoglobin solution (1 µM) and spectra were recorded over time using a UV-Vis spectrophotometer (Genesys 10S, Thermo Scientific) until total oxyhemoglobin reduction. The oxidation of oxyhemoglobin to methemoglobin by the presence of NO is monitored by calculating the differences in absorbance from the most intense peak over time, which allows the real-time quantification of the NO that is being released by the material. [2]

Briefly, this method monitors the NO release according to the following equation:

oxyHb + NO→metHb + NO_3

This reaction is faster than other competing reactants present in the medium (such as oxygen, superoxide ions, thiols, etc.), which makes the NO capture by oxyhaemoglobin almost stoichiometric in most experimental conditions.[2]

Four independent measurements were performed with different MIP-210 samples, giving a standard deviation from the average fluxes lower than 0.12 µM.

First-principles calculations: Spin-polarized periodic Density functional theory (DFT) calculations were performed to model the adsorption of NO in MIP-210(Fe) with Fe(III) saturated with terminal water molecule. All calculations were performed using the projector augmented wave (PAW) potential[3] as implemented in the Vienna Ab initio Simulation Package (VASP).[4] These calculations considered a 2x1x1 supercell of the MIP-210(Fe)-100K crystal structure free of adsorbed water. Similar calculations were performed for the crystal structure determined for the MOF filled of water molecules. To identify the transition states (TS), the climbing image bare elastic band (CI-NEB) method[5] was used and each located TS structure possesses only one imaginary frequency. A plane-wave basis set with a kinetic energy cut-off of 800 eV was used with Brillouin-zone sampling at gamma point. A force convergence criterion of 0.01 eV $Å^{-1}$(0.02 eV $Å^{-1}$ for CI-NEB calculations) and an electron self-consistent convergence criterion of $1×10^{-5}$ eV was consistently used.

Cell culture and cytotoxicity tests: HeLa cells (human cervical cancer cell line) were grown in RPMI-1640 medium supplemented with foetal bovine serum (10 % V/V), penicillin-

streptomycin (100 UI.mL^{-1} and 100 µg.mL^{-1}, respectively) and 2 mM glutamine. HEKn cells (epidermal keratinocytes isolated from neonatal foreskin) were cultivated in EpiLife® Medium supplemented with calcium, human keratinocyte growth supplement (HKGS) and an antibiotic ⁄ antimycotic solution of gentamicin and amphotericin B. HUVECs were cultured in Medium 200PRF supplemented with low serum growth supplement (LSGS). All cells were incubated at 37 °C in a humidified atmosphere with 5 % CO_2. Fresh medium was replaced every 1-2 days until the adequate confluency for subcultivation.

Viability/toxicity was estimated by the fluorometric resazurin assay. Cells were seeded in 96-well plates at the density of 7500 cells/well for 24 hours assays and 2500 or 5000 cells/well for 72 hours assays (HeLa or HUVEC/ HEKn cells, respectively). After 24 hours of incubation, the medium was replaced by fresh medium or by a suspension of medium with the sample at the desired concentration. Eight replicates were used for each condition. On the respective time, 10 µL per well of resazurin (final concentration of 10 µg/mL) was added and incubated for more 3 hours. Resazurin reduction was quantified by fluorescence (λ_{ex} = 530 nm, λ_{em} = 590 nm) in a Spectra Max Gemini EM reader from Molecular Devices. The fluorescence value of the supplemented medium was subtracted. Cell viability was calculated using the following equation:

Cell viability (%) = (F sample / F control) × 100

where F sample is the fluorescence of the cells incubated with samples and F control is the absorbance of the cells incubated without the sample.

HUVEC cells migration assay: HUVEC cell migration was evaluated using the Oris^{TM} Cell Migration Assay (Platypus technologies, LLC, Madison WI). In brief, HUVEC cells (50000 cells/well, from 2 to 5 passage) were seeded in a 96-well plate with "stopper" barriers in each well that create a central cell free detection zone. After incubation overnight, the stoppers were removed, allowing the cells to migrate into the detection zone, and the medium was replaced by fresh medium or by a medium containing unload or NO-loaded MIP-210(Fe) (11.75 µg.mL^{-1}). After 24 and 48 hours, each well was photographed using a microscope (Olympus, CK40) equipped with a digital camera (C4040; Olympus). A black mask with 96 prefabricated openings that precisely frame the central detection zone was attached to the bottom of the 96-well plate when the pictures were taken. The cell-free area within the detection zone was calculated using the ImageJ 1.50i software with a MRI wound healing tool [6]. The migration rate after 24 and 48 hours was calculated using the following equation (1):

$$\% \, migration \, rate = \frac{(Area_{pre-migration\ (t=0\ h)} - Area_{post-migration\ (t=x\ h)}}{Area_{pre-migration\ (t=0\ h)}} \cdot 100$$

The average percentage of migration rate and standard deviation of at least three independent assays (with ≥3 replicates each) were reported in the results. Unpaired student's t-test was used to assess significance with p-values < 0.05 considered statistically significant.

Endothelial cell tube formation assay: To confirm the angiogenic properties of the NO released by the MIP-210(Fe), tube formation assays were conducted using Geltrex™ as basement matrix and HUVEC cells (between 2-5 passage). Pre-frozen Geltrex™ (4ºC overnight in an ice bath) was added to a 24-well plate (95 µL/well) and the precoated plate was incubated for 30 minutes at 37 ºC to allow the gel to solidify. Then, HUVEC cells (225000 cells mL^{-1}) were diluted in supplemented Medium 200PRF in the absence or presence of the unloaded or NO-loaded MIP-210(Fe) and seeded in each gel-coated well (85500 cells/well). The plate was incubated at 37 ºC, 5% CO_2 for 18 hours. After that period, the tube network formation was observed and photographed with a microscope (Olympus, CK40) equipped with a digital camera (C4040; Olympus). The number of microvascular branches formed was quantified using ImageJ 1.50i software with the angiogenesis analyser plugin. Quantification data presented is expressed in comparison with the control. Three independent assays with 3 replicates each were performed for each condition. Unpaired student's t-test was used to assess the statistical significance.

Section 2 - MOF characterization

SEM images evidence that parallelepiped-shaped single crystals are formed. The size distribution of MIP-210(Fe) synthesized in a glass vial is uneven as shown in Fig S1a. The particles synthesized in a Teflon reactor are more uniform with a size around 1µm (Fig S1b). After activation at 120°C for three hours under vacuum, some particles were broken into smaller particles (Fig S1c and S1d). Single crystals for diffraction were obtained through putting the reaction solution in a Teflon reactor at 180°C for 72h (Figure S2).

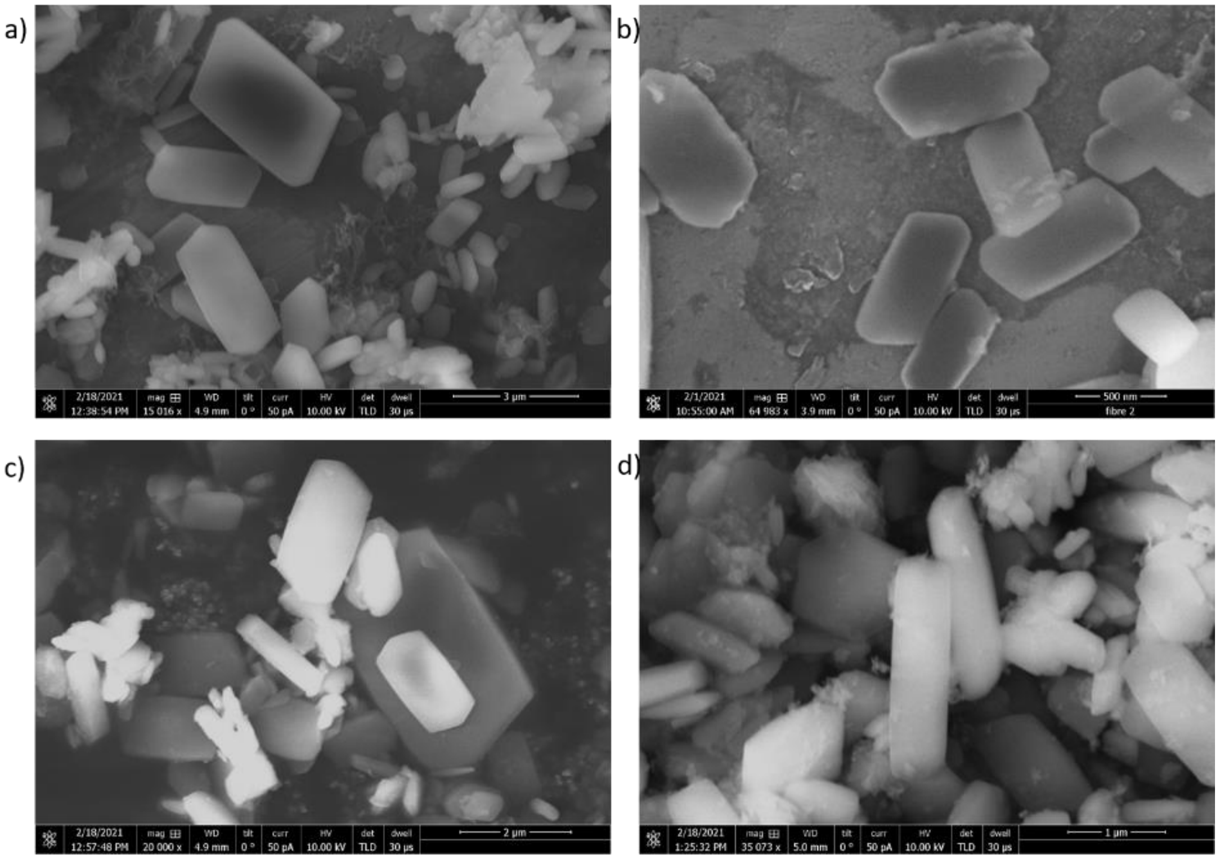

Figure S1. SEM images of MIP-210(Fe) as synthesized (left) and after activation (right) in a glass bottle (a and c) and in a Teflon reactor (b and d).

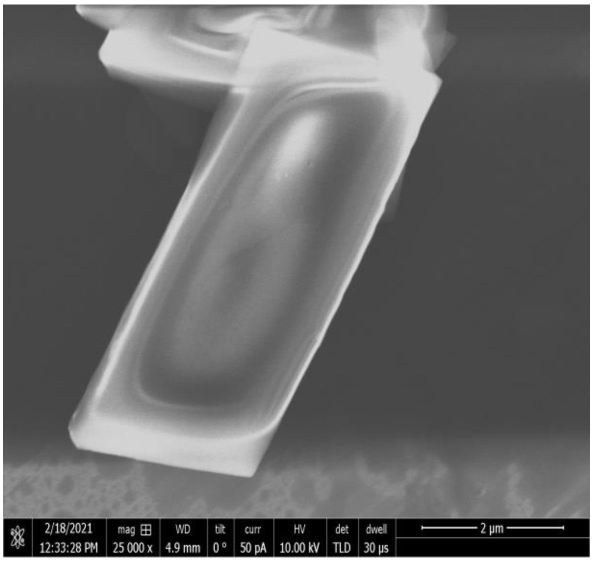

Figure S2. SEM image of a MIP-210(Fe) single crystal prepared at 180°C.

The EDX analysis shows an average phosphonate/iron molar ratio of 65/35, which corresponds to the formula (1:1) of MIP-210(Fe) (see Table S1) or $Fe^{III}(H_2O)[O_3P\text{-}CH_2\text{-}C_6H_4\text{-}CH_2\text{-}PO_3H].H_2O$.

Table S1. EDX data corresponding to the SEM images in Figure S1.

Atomic percentage	MIP-210(Fe) Fig S1a	MIP-210(Fe) Fig S1b	MIP-210(Fe) Fig S1c	MIP-210(Fe) Fig S1d
P	64.72	66.67	65.36	66.82
Fe	35.28	33.33	34.64	33.18

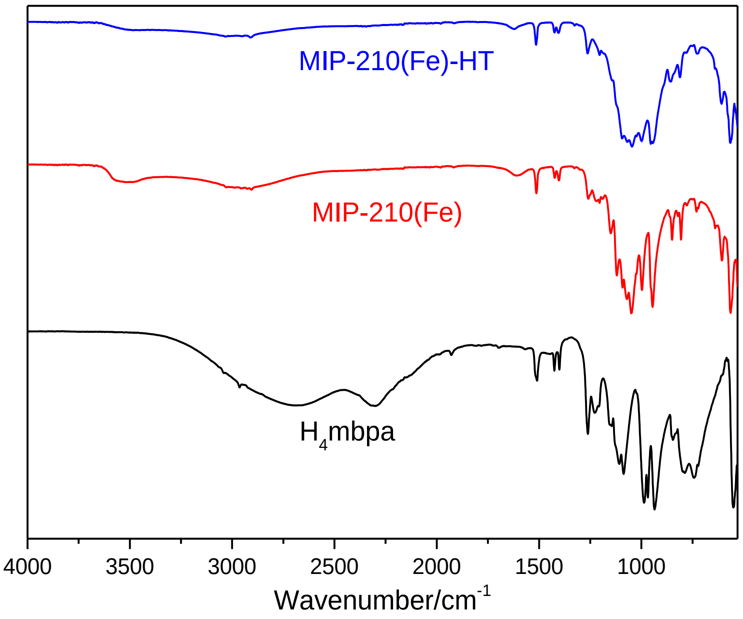

Figure S3. Infrared spectra of H_4mbpa and MIP-210(Fe) at 293 K before and after thermal treatment.

2.1. Structure Determination

The structure of MIP-210(Fe) was solved using synchrotron-based single crystal X-ray diffraction data collected with a microfocused bean on Proxima 2A beamline (Synchrotron SOLEIL) from a suitable single crystal. It was found that MIP-210(Fe) [$FeO(C_8H_9O_6P_2)$•H_2O or $Fe^{III}(H_2O)[O_3P\text{-}CH_2\text{-}C_6H_4\text{-}CH_2\text{-}PO_3H]\cdot H_2O$] crystallizes in a monoclinic $P2_1/n$ space group with the unit-cell parameters of a = 5.1440(3)Å, b = 10.7106(7)Å, c = 21.06536(9)Å and β = 93.222(4) °. The simulated PXRD pattern matches well with the experimental powder pattern, which confirms the phase purity (Figure S4).

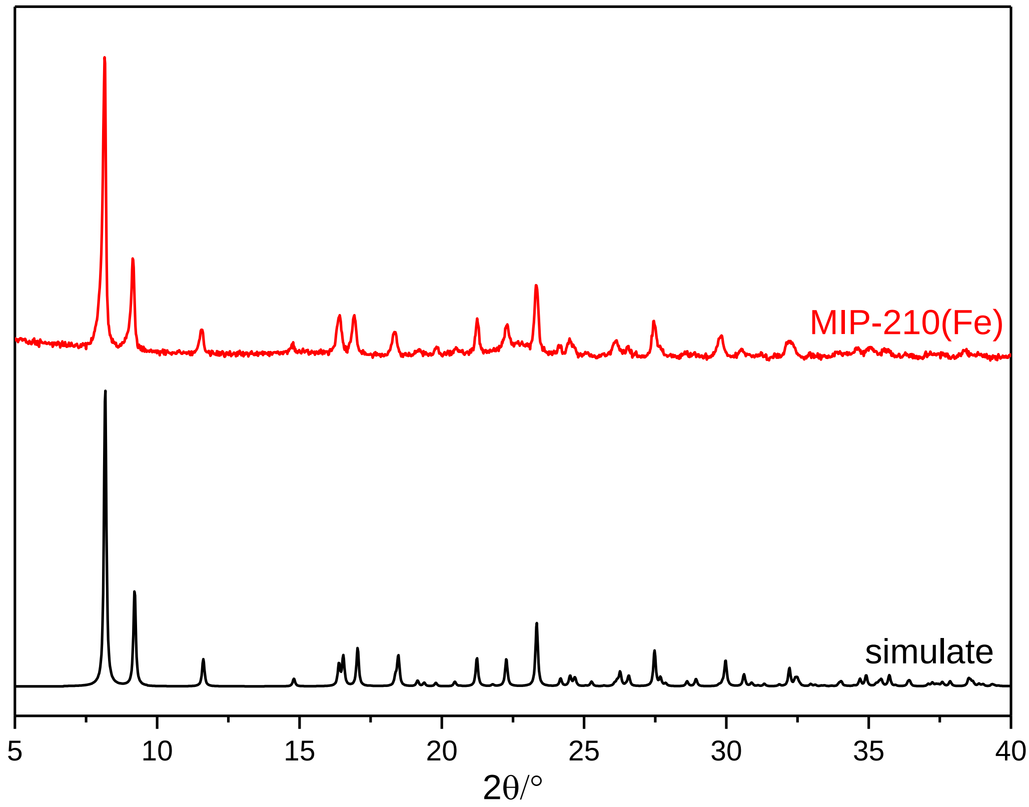

Figure S4. PXRD pattern (λ=1.5418 Å) of as-synthesized MIP-210(Fe) compared with the simulated PXRD pattern calculated from the single crystal structure.

a)

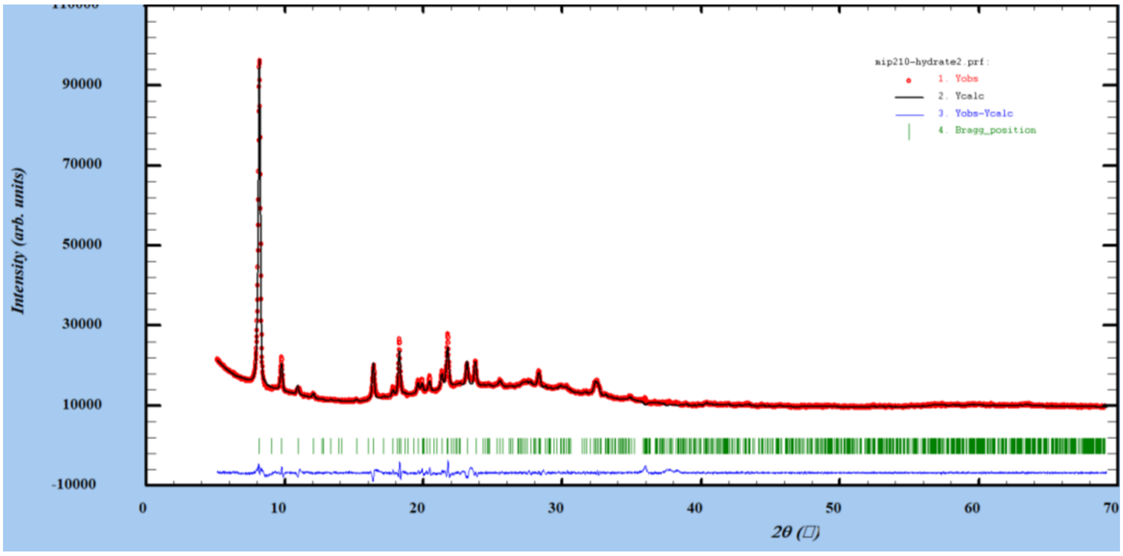

b)

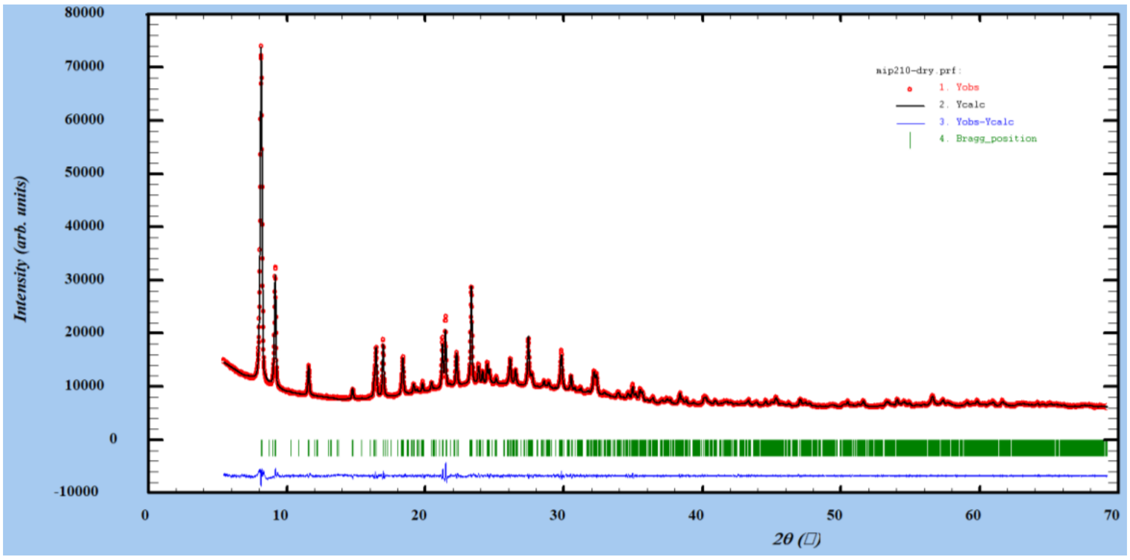

Figure S5. The Le Bail plots of a) as prepared (hydrated) and b) dried (after thermal treatment at 150°C) samples of MIP-210(Fe)

The HR PXRD shows the extra peaks for all the samples compared with the single crystal structure, which suggests the doubling of the parameter *a*, if compared with the initial single crystal model. This can be explained by the fact that the measurements are performed at slightly different temperature (100 K for single crystal, 293 K for powder).

Table S2. The MIP-210(Fe) unit-cell parameters obtained from the Le Bail refinements.

	**As-synthesized MOF**	**dried MOF**	**NO-loaded MOF**
Space group	$P2_1/n$	P-1	$P2_1/n$
a (Å)	9.9729(5)	10.2648(2)	9.9550(9)
b (Å)	9.9587(5)	10.7643(2)	9.968(1)
c (Å)	22.3520(8)	22.0510(5)	22.271(3)
α (°)	90	90.150(1)	90
β (°)	104.704(5)	100.215(1)	104.673(4)
β (°)	90	89.960(2)	90
V (Å^3)	2147.2	2397.8	2138.0

Table S3. Characteristic bond distances of the MIP-210(Fe) structure.

**Bond**	**Distance / in Å**
Fe-O004 (R-PO3)	1,991
Fe-O005 (R-PO3)	1,956
Fe-O008 (R-PO3)	1,956
Fe-O006 (R-HPO3)	2,032
Fe-O007 (R-HPO3)	2,007
Fe-O009 (H2O)	2,113

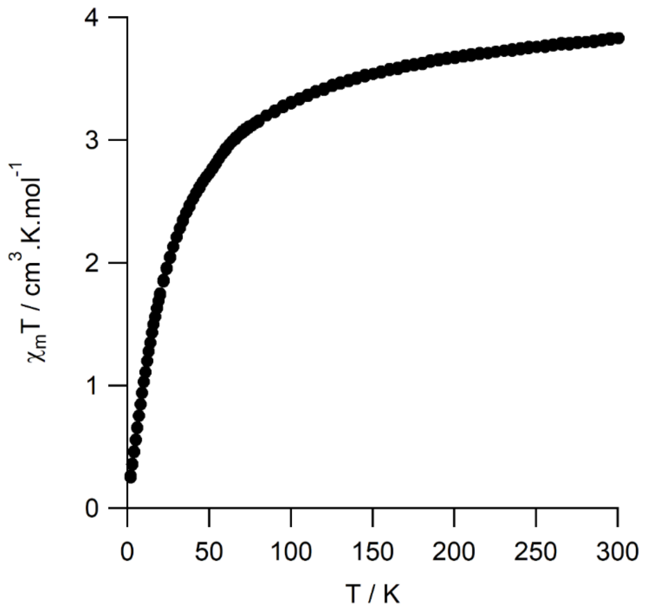

Figure S6: Evolution of the product of the molecular magnetic susceptibility by the temperature as function of the temperature measured on MIP-210(Fe) powder (measurement performed under an external magnetic field of 1000 Oe).

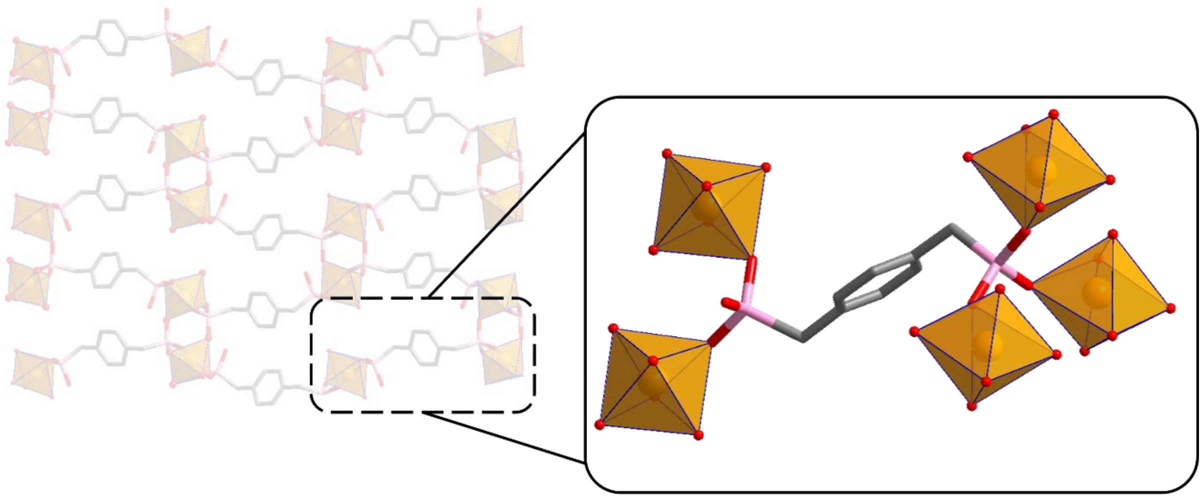

Figure S7. Crystal structure of MIP-210(Fe), where all binding sites of p-xylenediphosphonic acid are shown. As can be observed, one phosphonate group is fully deprotonated (coordinated with three adjacent Fe(III) cations) while the other is partially deprotonated (coordinated with only two adjacent Fe(III) cations). Color code: $FeO_5(H_2O)$, dark yellow polyhedra; O, red; C, grey; P, pink. Hydrogen atoms and non-coordinated guest molecules have been omitted for clarity.

2.2. MOF thermal and chemical stability

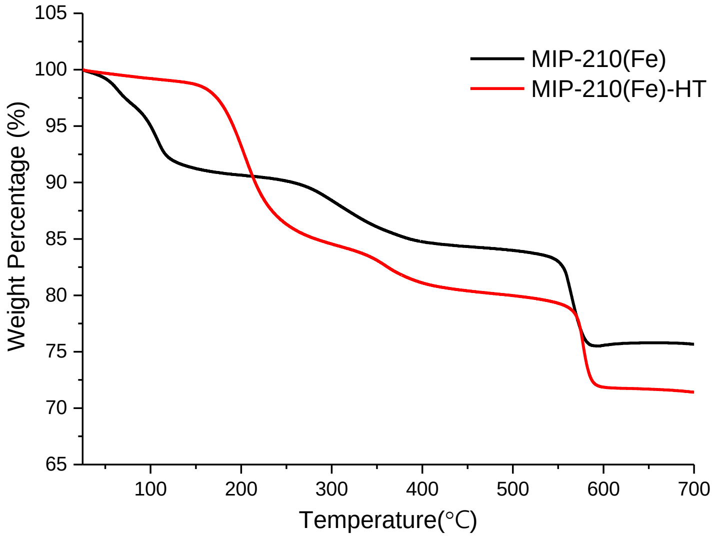

Figure S8. Thermogravimetric analysis of pristine and thermally treated MIP-210(Fe).

As observed in Fig. S8, pristine sample (MIL-210(Fe) or $Fe^{III}(H_2O)[O_3P-CH_2-C_6H_4-CH_2-PO_3H].H_2O$) started to lose weight immediately upon heating. The first weight loss (8.6 wt%) from room temperature to 160 °C could be ascribed to the losses of guest water molecules. Then the weight loss slowed down to reach a plateau, which was followed by the second (160 to 350 °C) and the third weight (500 to 700 °C) losses. The second weight loss could be ascribed to the removal of the coordinated water molecules, whereas the third one corresponds to the degradation of the ligand. In contrast, MIP-210(Fe)-HT compound shows very limited weight loss from the beginning until 160°C, as the activation progress almost remove all guest water molecules in the pore.

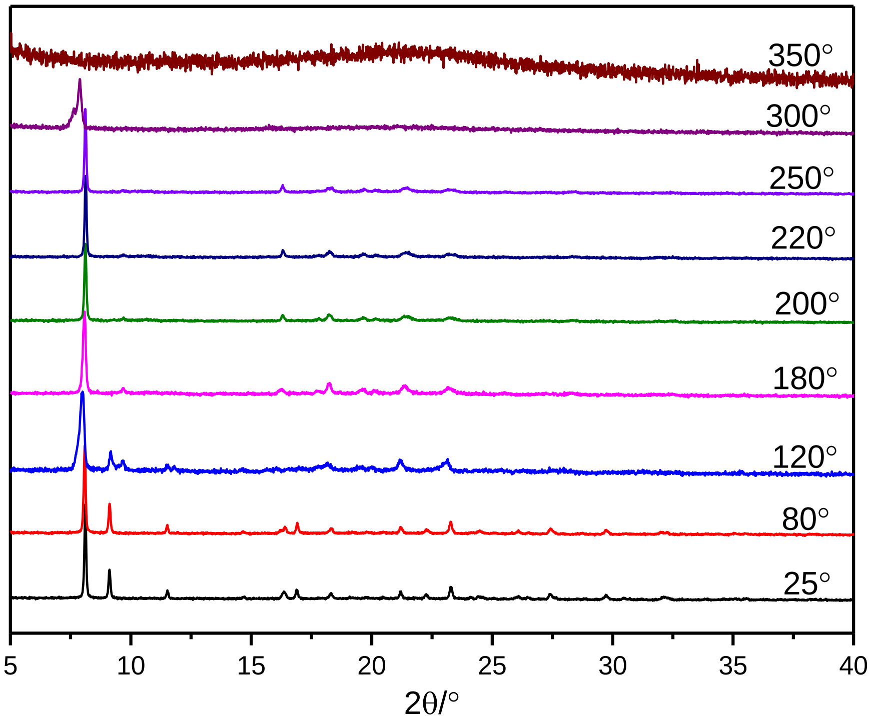

Figure S9. Variable temperature powder X-ray diffraction patterns (λ=1.5418 Å) of MIP-210(Fe).

As observed in Fig. S9, phase change could be observed through variable temperature XRD after 120°C that may be caused by the guest water molecules removal. The structure collapsed after 300°C.

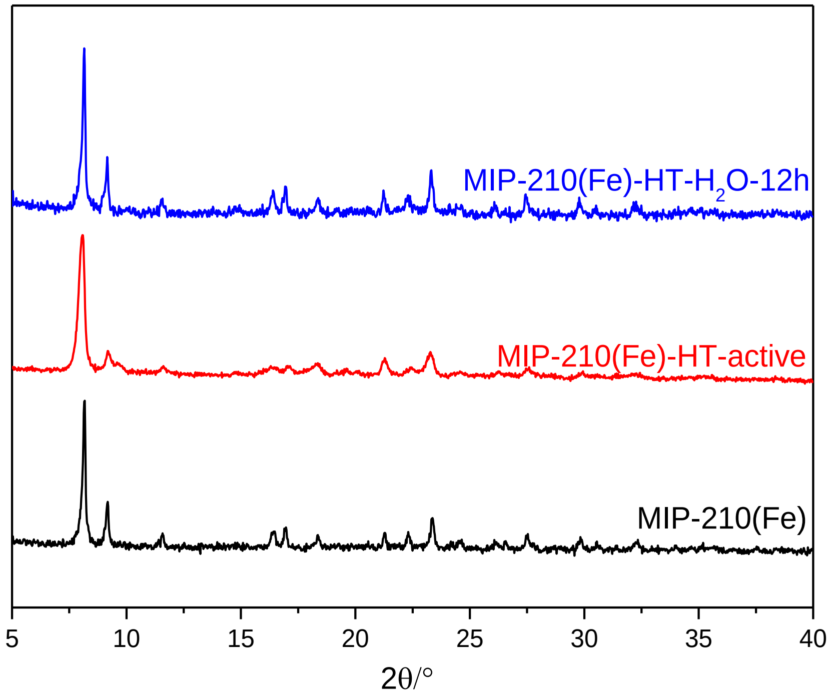

Figure S10. PXRD patterns (λ=1.5418 Å) of MIP-210(Fe) before, after thermal treatment under vacuum at 120°C and after soaking the thermally treated powder in water for 12h.

MIP-210(Fe)-HT can be obtained by thermal treatment under vacuum at 120°C. The starting phase can be restored by soaking the powder in water for 12 hours as shown in Fig S10.

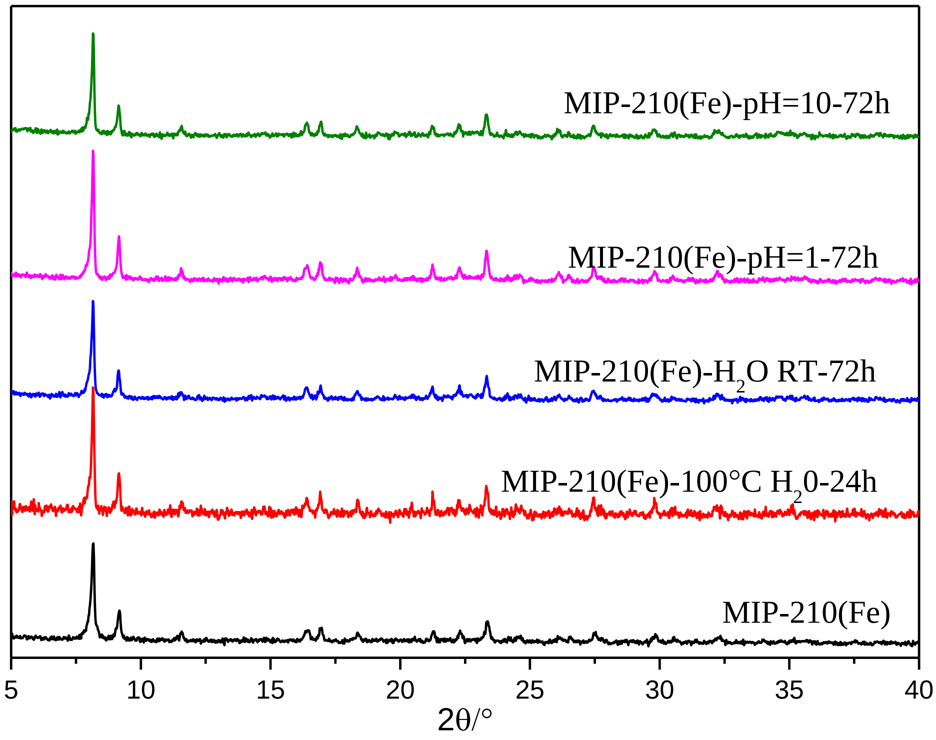

Figure S11. PXRD patterns (λ=1.5418 Å) of MIP-210(Fe) soaked in water at different pH.

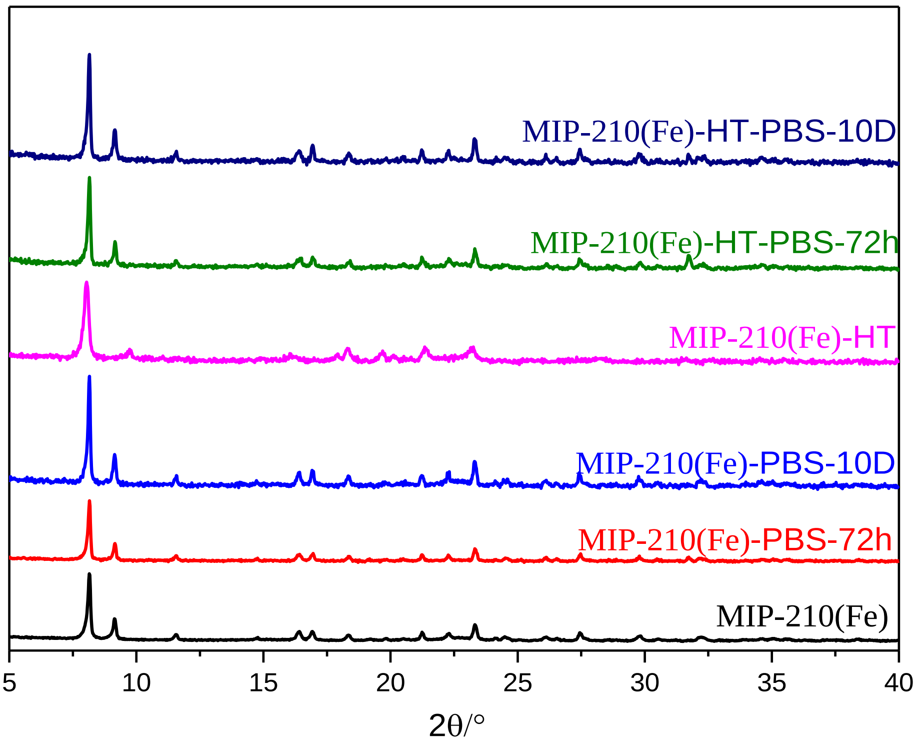

Figure S12. PXRD patterns (λ=1.5418 Å) of MIP-210(Fe) soaked over 72 h and 10 days in PBS (pH=7.4).

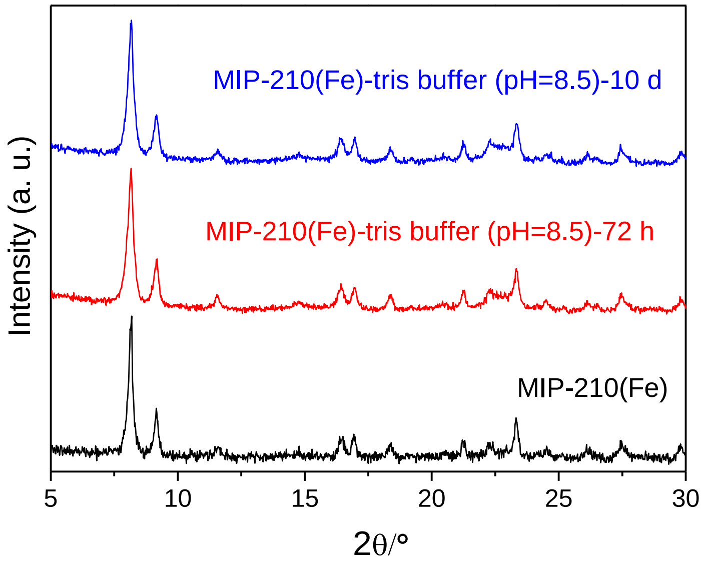

Figure S13. PXRD patterns (λ=1.5418 Å) of MIP-210(Fe) soaked over 72 h and 10 days in Tris buffer (pH=8.5).

For the stability test in PBS and Tris, 4 mg of MOF powder was soaked in 4 mL of buffer and the mixture was kept at 37 °C in an oven under agitation.

2.2.1 Stability tests in biological conditions

Table S4. Leaching metal percentage according to the amount of metal present in the MOF quantified in different biological media after incubating MIP-210(Fe) for 72 h under biological conditions. The stability of MIL-100(Fe), MIL-127(Fe) and MIP-177(Ti) has been investigated under the same conditions for comparison.

**Material**	**MIL-100(Fe)**	**MIL-127(Fe)**	**MIP-177(Ti)**	**MIP-210(Fe)**
RPMI-1640 medium	6.36	2.20	0.40	0.87
EpiLife® medium	2.45	1.37	0.87	0.68
Medium 200PRF	0.31	1.25	<0.08	0.57

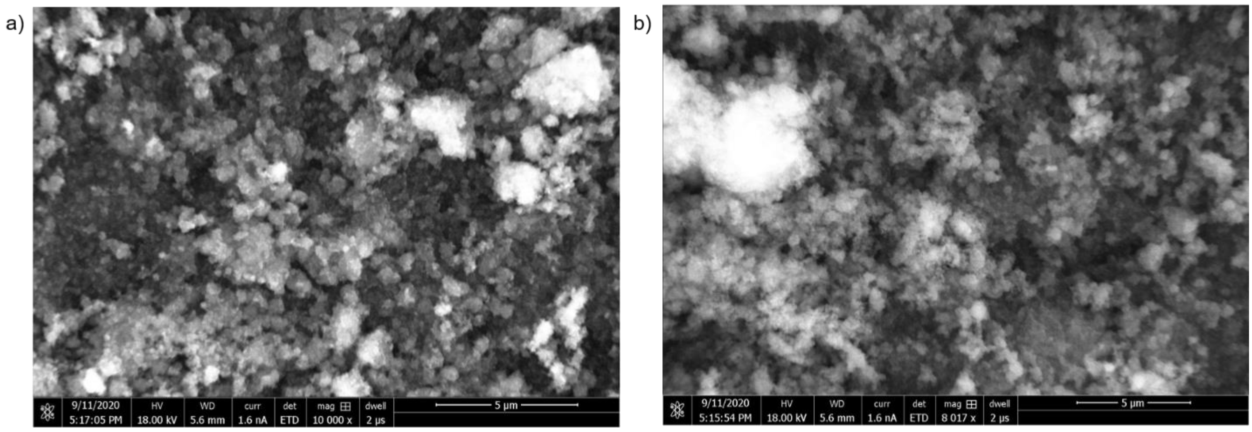

Figure S14. SEM images of the MIP-210(Fe) after being incubated for 72 h in serum-supplemented RPMI-1640 medium (a) and serum-supplemented Epilife® medium (b). Images were obtained on the dry powder after being washed with water.

Table S5. EDX data corresponding to Figure S10

Atomic percentage	MIP-210(Fe) Fig S1a	RPMI-1640 medium Fig S11a	Epilife® medium Fig S11b
P	64.72	63.59	64.07
Fe	35.28	36.41	35.93

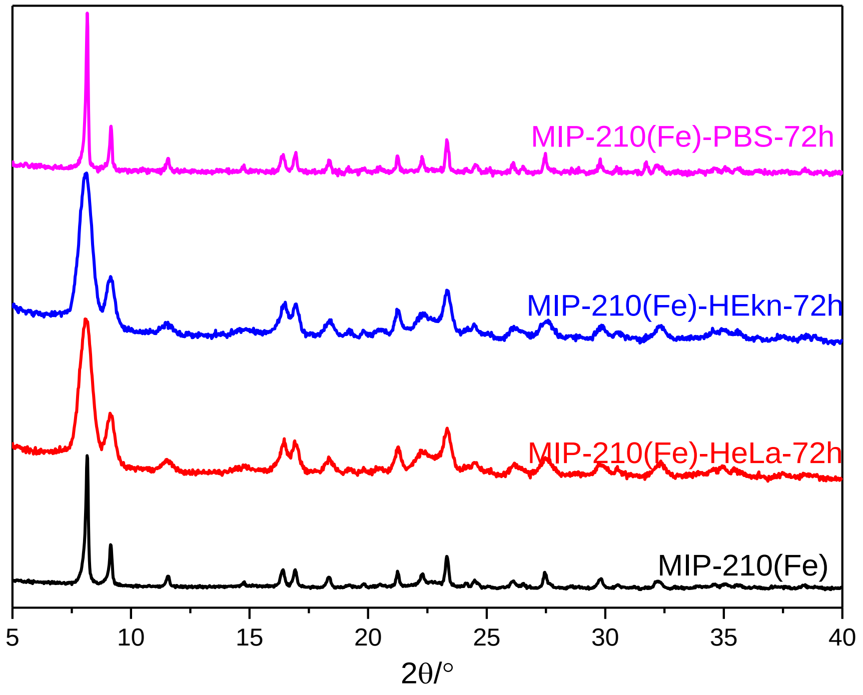

Figure S15. PXRD patterns (λ=1.5418 Å) of MIP-210(Fe) before and after being incubated for 72 h in PBS (pink), serum supplemented RPMI-1640 medium (used for HeLa culture) (red) and

serum-supplemented Epilife® medium (HEKn cells medium) (blue). The PXRD patterns were obtained on the dry powder after washing with water and centrifugation.

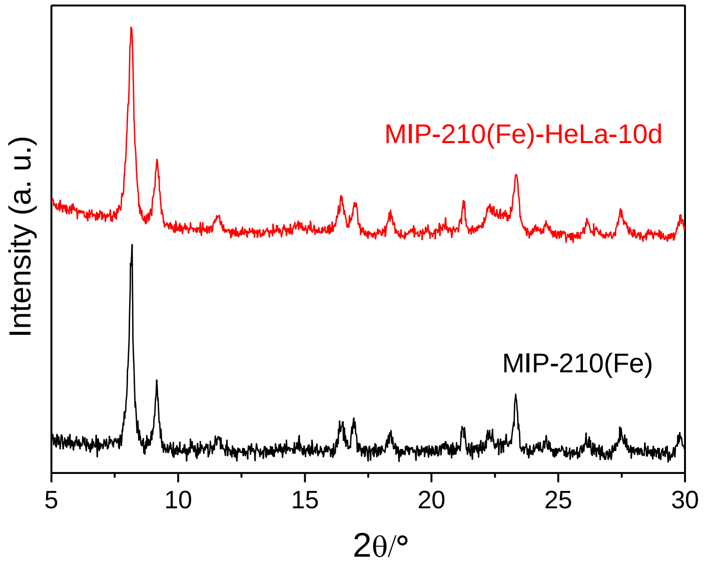

Figure S16. PXRD patterns (λ=1.5418 Å) of MIP-210(Fe) before and after being incubated for 10 days in serum supplemented RPMI-1640 medium, confirming the high stability of MIP-210(Fe) over extended periods. The PXRD pattern was obtained on the dry powder after washing with water and centrifugation.

Table S6. Leaching iron percentage after contact with Medium200PRF at different times at 37 ºC.

**Contact time**	**24 h**	**48 h**	**72 h**
Iron content (µg/mL)	0.31	0.41	0.57

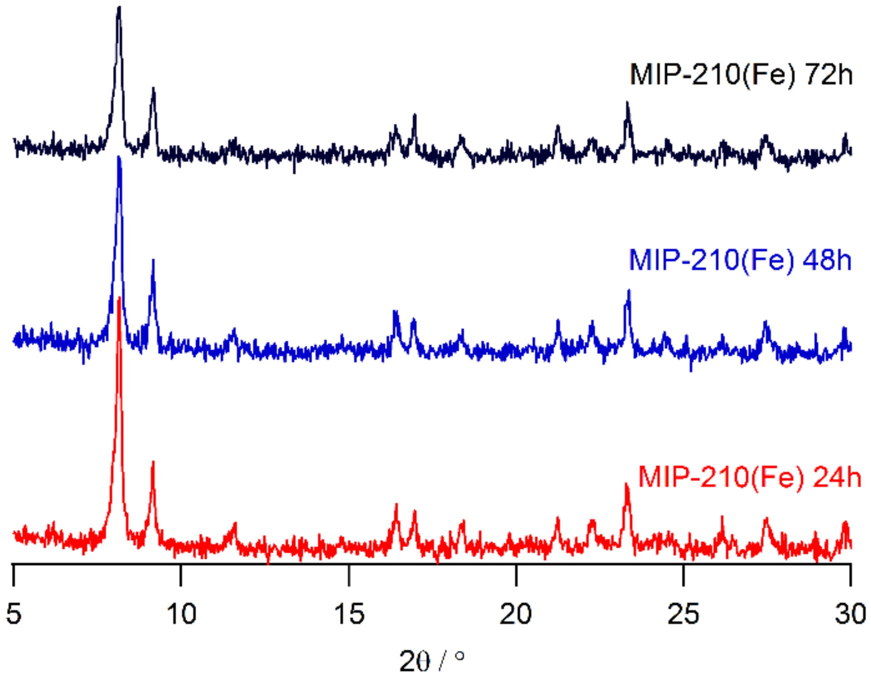

Figure S17. PXRD patterns (λ = 1.5418 Å) of MIP-210(Fe) after being incubated for different times (24 h, 48 h and 72 h) in serum supplemented Medium 200 PRF (used for HUVEC culture).

2.3. Detailed discussion on MIL-210(Fe) stability

Prior considering any new delivery platform for medical purposes, their stability in physiological media and biocompatibility must be taken into consideration at early stages. This is a prerequisite as these nanomaterials need to remain intact over at least the period they are delivering the therapeutic agent and do not induce any toxicity over the target tissues. MOFs sometimes presents toxicity issues since they hold organic acids and metals (toxic, in many cases) and they are commonly less thermally and chemically stable than porous inorganic materials (e.g., zeolites)[7,8]. For instance, CPO-27 (Ni or Co) and HKUST-1 exhibit the highest NO adsorption capacities, but they are not therapeutically relevant as leaching of toxic metals occurs once they degrade in aqueous solutions[9,10]. On the contrary, most iron MOFs like MIL-88, MIL-100 and MIL-127, as well as CPO-27(Mg, Zn) do not present strong toxic impact once in contact with cells[11–13]. However, nanoparticles of these MOFs are not stable enough in biological media with a degradation kinetics of typically a few hours, which compromises the control of the NO release, particularly if a long-term therapeutic delivery is required. Remarkably, MIP-210(Fe) is highly stable in aqueous media from pH=1 to pH=10 and it does not present any degradation after soaking ten days at 37°C in pH=7.4 PBS solution and Tris buffer (pH=8.5) (Fig. S12-S13). The stability under physiological conditions was evaluated in three representative biological media, RPMI-1640, Medium 200PRF and Epilife, all supplemented with serum. These media support the culture of a wide range of mammalian cells, including the cells used in this work. After incubation in RPMI-1640, Epilife and Medium 200PRF for 72h, the PXRD patterns of the incubated material did not present noticeable changes (Fig. S15 and S17) and the % Fe leached in the liquid was 0.87, 0.68 and 0.57, respectively, indicating minor MOF degradation. Of note, PXRD pattern of the material after being soaked ten days in RPMI-1640 confirms the crystallinity remains intact even after longer periods (Figure S16). This stability is comparable with the one of MIP-177-LT, a $Ti_{12}O_{15}$ oxocluster tetracarboxylate MOF of interest for NO release application and much better than previous iron MOFs studied for NO adsorption (e.g., MIL-100(Fe) and MIL-127(Fe); Table S4)[14]. Besides, the EDX and SEM also confirmed this result (Table S5) albeit a slight crystal size reduction can be observed from SEM, most probably due to biological adaptability due to the breaking of the crystals into smaller particles (Fig. S14).

Section 3 - NO adsorption/release detail

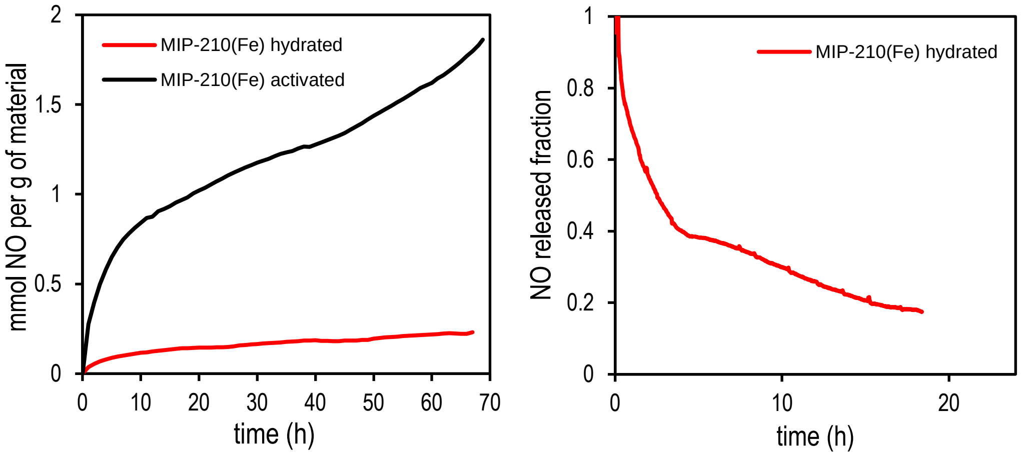

Figure S18. Adsorption/desorption kinetics of NO in the hydrated material (*i.e.* not activated) and adsorption comparison with the activated sample.

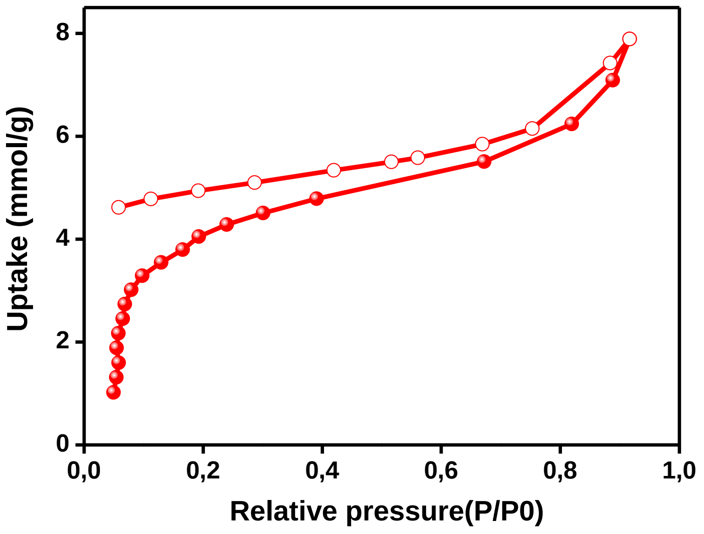

Figure S19. Water adsorption isotherm of MIP-210(Fe) at 25 °C confirms that water can enter in the pores and replace the coordinated NO. Adsorption and desorption isotherms are represented by full and empty symbols, respectively. The volumetric water adsorption measurements were collected using a Micromeritics-triflex instrument. Prior to the measurement, the sample was degassed using a Micromeritics SmartVacPrep degas unit.

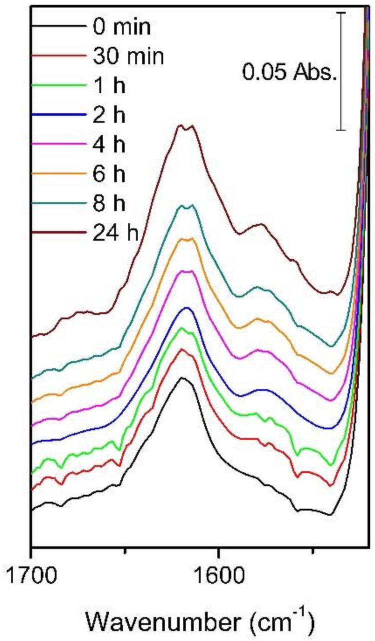

Figure S20. IR spectra of MIP-210(Fe) (previously activated at 120 ºC, 12h) at room temperature before and after NO exposure (P_{NO} = 1.3 kPa) for different times.

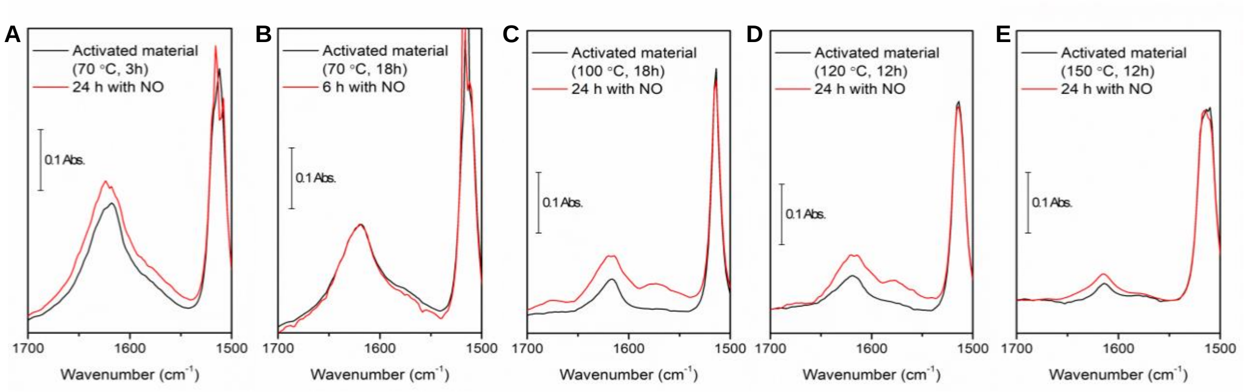

Figure S21. IR spectra of MIP-210(Fe) activated at different temperatures and further exposure to NO.

Several activation temperatures were used to understand the influence of the water adsorbed in the pores on the NO adsorption. According to the TGA and VT-PXRD measurements (Figure S8 and S9, respectively), the solid is thermally stable up to 250 ºC. After, heating the sample at 70 ºC for 3 hours (Fig. S21a), a reduction of the water absorbance band intensity (ca. 1630 cm^{-1}) was noticed, which was more pronounced when heated for a longer period (18 hours) (Fig. S21b). However, in both cases, no NO adsorption was observed, suggesting that there is still a considerable amount of physisorbed water molecules that impedes NO from entering the pores. By activating the material at 100 ºC and 120 ºC (Fig. S21c and d), a significant reduction in the water absorption band was observed and a new NO adsorption band appeared. It indicates that the physisorbed water was removed by the thermal treatment, therefore allowing NO to access the pores and to replace some of the chemisorbed water on the OMS. By increasing the

activation temperature to 150 ºC (Fig. S21e), no NO adsorption was observed. This observation together with the VT- XRD suggesting that at this temperature, a slight pore contraction occurs associated to the full removal of physiosorbed water (see Fig. S9), which prevents the access of the NO in the ultra-narrow pores. Thus, activation temperatures between 100 ºC and 120 ºC seem to be in the most indicated range for a more effective NO adsorption.

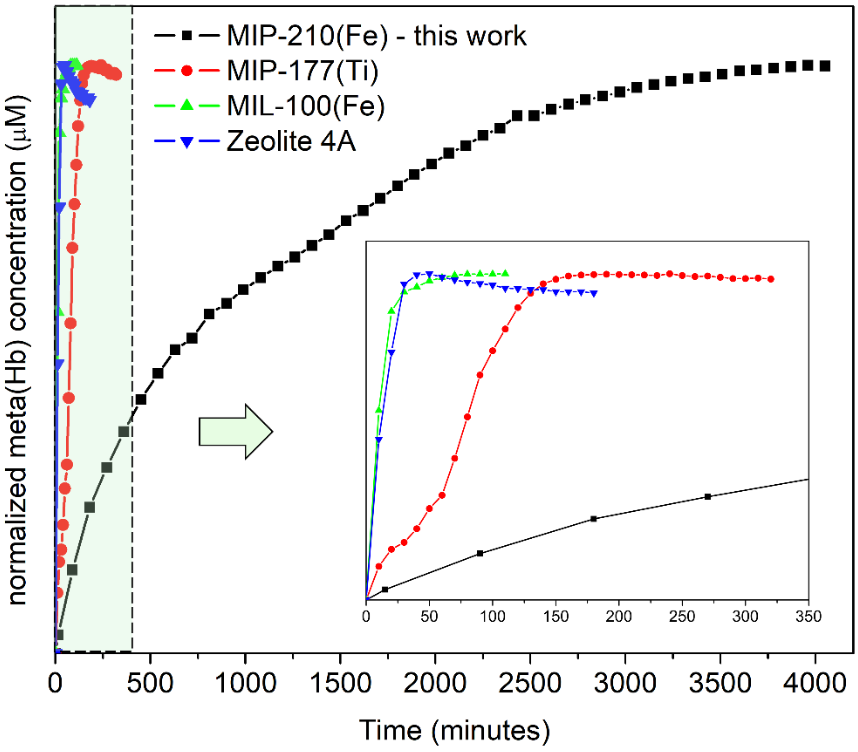

Figure S22. Comparison of NO release profiles between MIP-210 and other reported porous materials (MIL-100(Fe), MIP-177(Ti) and Zeolite 4A) using the oxyhemoglobin quantification assay at 25 °C. The concentration of meta(Hb) quantified is considered stoichiometric to the concentration of NO released[2]. This value was normalized for clear comparison. The inset graph zooms in on the profiles within the green bar, focusing on the x-axis range of 0 to 350 minutes. As can be seen in the case of MIL-100 and Zeolite 4A, all the oxyhaemoglobin is consumed in the first minutes which means there is a fast release of high NO amounts, as a consequence of lack of biological stability and absence of chemisorption combined with high water affinity, respectively. In the case of MIP-177, the release is more controlled but due to its large pore size (1.1 nm), the water diffusion is too fast, with a subsequent release of NO that does not last more than 2 hours. Of note, when the total meta(Hb) quantification reaches a plateau, it indicates that the oxy(Hb) in the medium has been completely consumed. This highlights that quantification is only comparable during the consumption period of oxy(Hb).

a)

b)

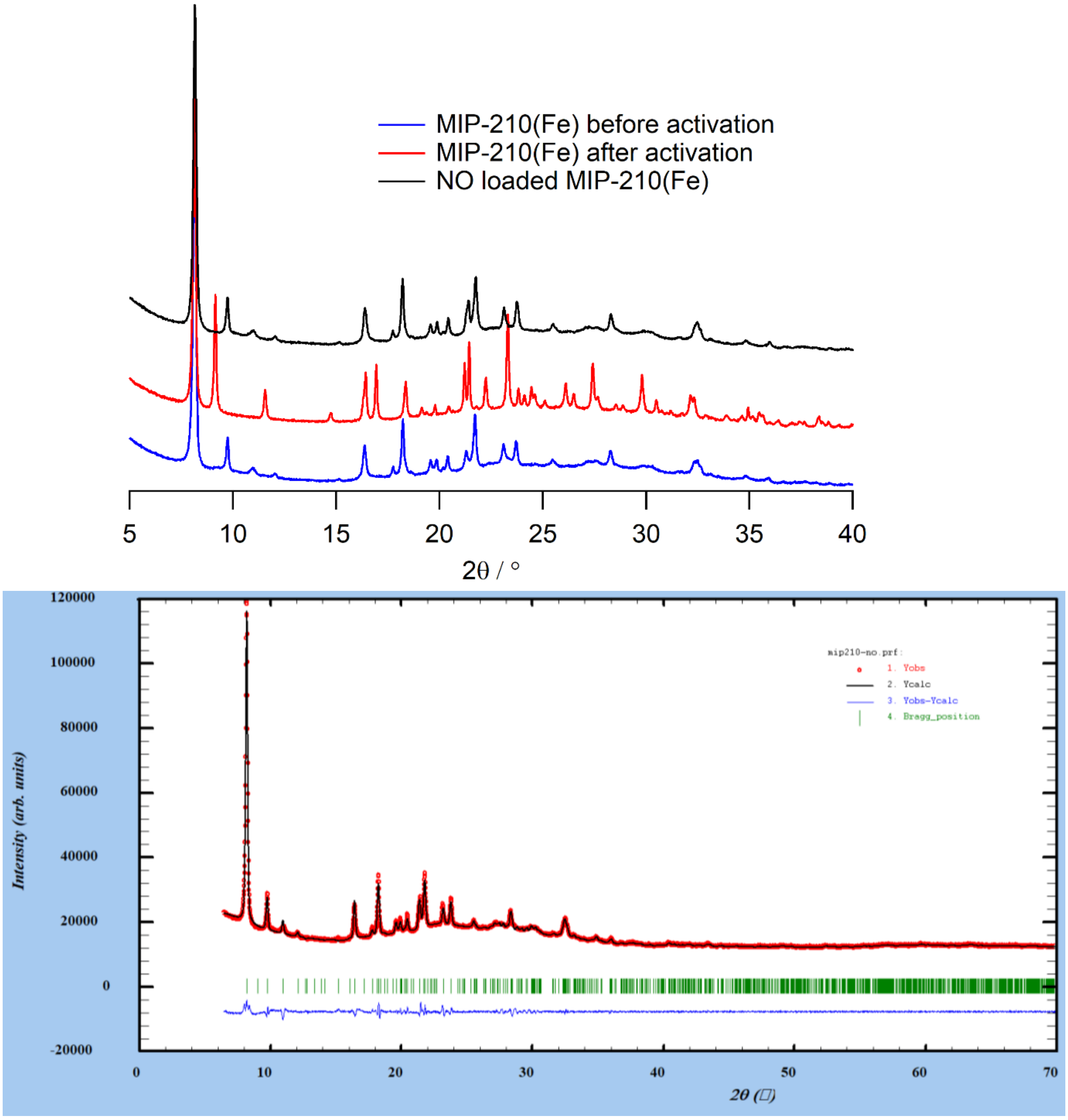

Figure S23. a) PXRD patterns (λ=1.5418 Å) of MIP-210(Fe) before activation, after activation at 120° for 2 hours and after loading the activated sample with NO; b) Le Bail plot of NO-loaded MIP-210(Fe).

3.1. Molecular simulation

As a preliminary stage, the coordinated water molecules were removed from the free adsorbed water MIP-210(Fe)-293(K) to explore the most stable Fe spin state configuration. All ferromagnetic (FM), ferrimagnetic (FiM) and antiferromagnetic (AFM) configurations have been tested for the magnetic ground state and further DFT-optimized (see Figure S24) using the projector augmented wave (PAW) [15] formalism within the generalized gradient approximation (GGA) method with Perdew-Burke-Ernzerhof (PBE) exchange-correlation functional as implemented in Vienna Ab Initio Simulation Package (VASP) [3,16,17]. The convergence criteria of 0.01 eV/Å(0.02 eV/Å for transition state searching) and 10^{-5} eV were used for the forces and energy convergence during the optimization process, respectively. The DFT-D3 method [18] was implemented to include the dispersion contribution. The cutoff energy for the plane-wave basis set has been consistently set to 900 eV. The DFT+U approach was applied to treat the electron correlation of highly localized iron *d* orbitals, with the values of $U–J$ set to 4.0 eV [19]. These calculations evidenced that the Intra-chain AFM2 configuration is the most stable one (Table S7).

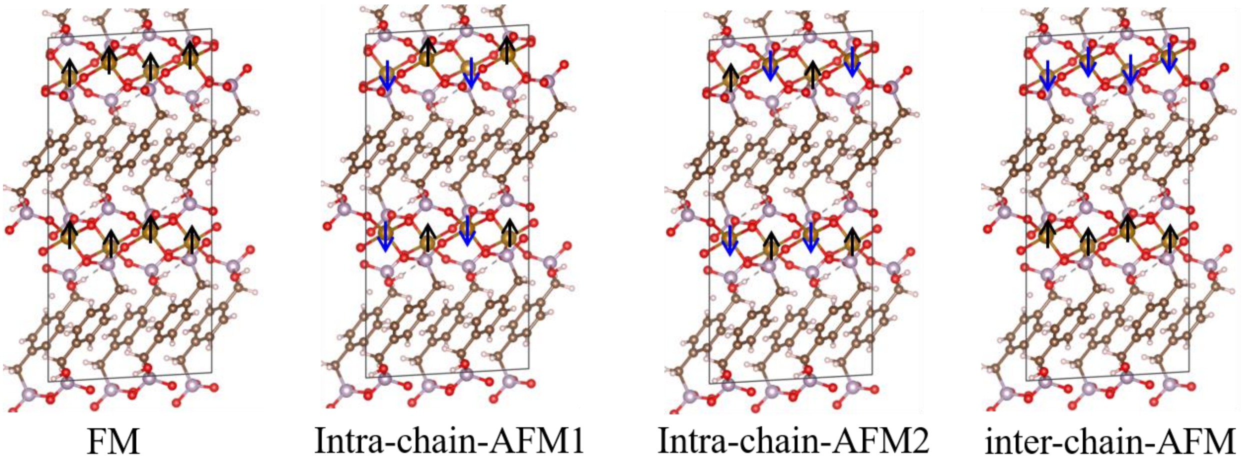

Figure S24. Different Fe spin states configurations of MIP-210(Fe). Iron, phosphorus, oxygen, carbon, and hydrogen atoms are in dark yellow, lavender, red, brown, and white respectively.

Table S7. Magnetic moment and relative energy (E_{rel}) for the different Fe spin states configurations of MIP-210(Fe)

	Magnetic moment/μB	E_{rel}/kJ mol^{-1}
FM	40	6.83
FiM	34	191.59
Intra-chain-AFM1	0	0.01
Intra-chain-**AFM2**	**0**	**0.00**
inter-chain-AFM	0	6.79

This most stable Intra-chain-**AFM2** configuration with 1 coordinated water to each Fe(III) was further loaded by 1 NO, for the adsorption and co-adsorption study respectively and equally DFT-optimized using the same functional mentioned above.

The adsorption energy of NO/NO+H_2O in MOF was calculated as:

$$E_{ads}(NO)=E(MOF+NO)-E(MOF)-E(NO) \quad (1)$$

$$E_{ads}(NO+H_2O)= E(MOF+NO+H_2O)-E(MOF)-E(NO)-E(H_2O) \quad (2)$$

where E(MOF+NO) and E(MOF+NO+H_2O) are the total energies of MOF loaded by 1 NO and 1 NO+1 H_2O molecule, respectively; E(MOF), E(NO) and E(H_2O) are the total energies of empty MOF, NO and H_2O molecule, respectively.

The transition state between was located using the climbing image nudged elastic band method (CI-NEB) [5] as implemented in the Transition State Tools for VASP (VTST) module [20].

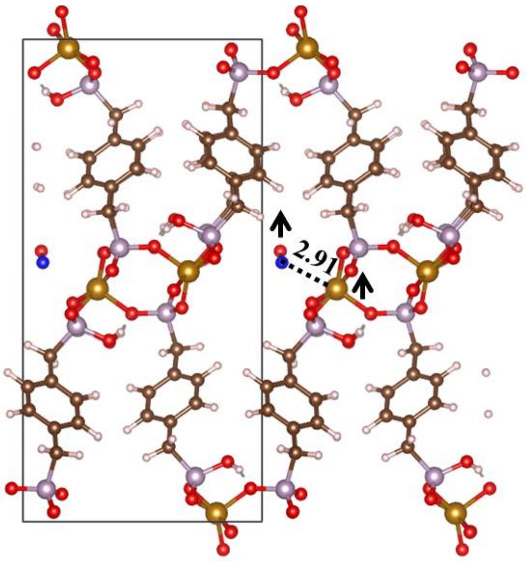

Figure S25. The adsorption configurations of spin-parallel (left) and spin-antiparallel (right) aligned in MIP-210(Fe), respectively. iron, phosphorus, oxygen, carbon, nitrogen and hydrogen atoms are in dark yellow, lavender, red, brown, blue and white respectively. The distances are in Å.

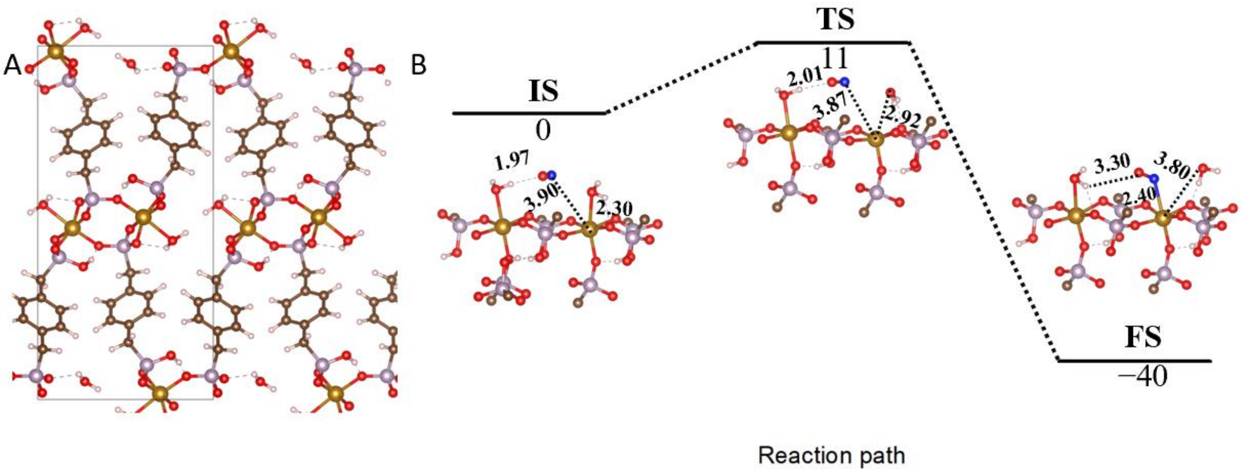

Figure S26. A) Side view of the fully hydrated structure of MIP-210(Fe); B) the potential energy profile of the displacement reaction of H_2O by NO in hydrated crystal structure. Color

codes: carbon (dark brown), hydrogen (white), oxygen (red), phosphorus (purple), nitrogen (blue) and iron (light brown). The distances and energies are in Å and kJ mol-1, respectively.

Section 4 - Cell culture and *in vitro* studies

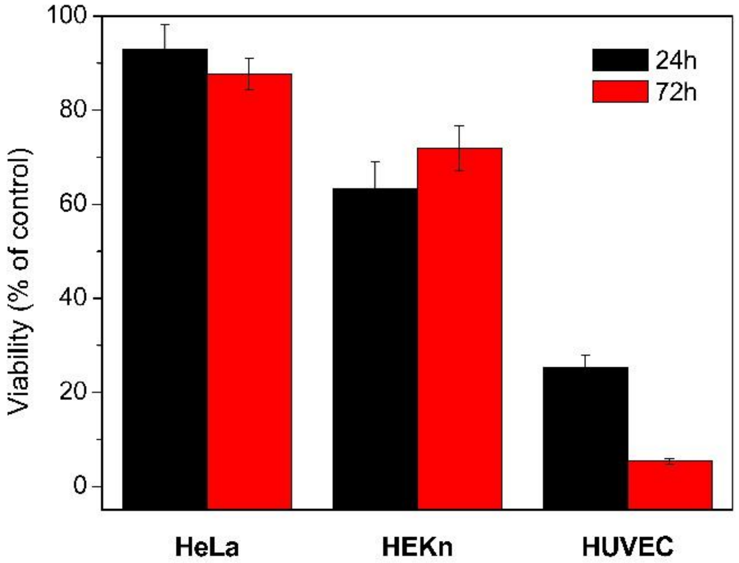

Figure S27. Cell viability of human cervical carcinoma cells (HeLa), primary keratinocytes (HEKn) and human umbilical vein cells (HUVEC) after 24 h and 72 h incubation with MIP-210(Fe) (not loaded with NO) at 450 µg.mL^{-1}. Error bars represent the standard deviation of eight replicates.

Cell viability (%) = (F sample / F control) × 100

where F sample is the fluorescence of the cells incubated with samples and F control is the absorbance of the cells incubated without the sample.

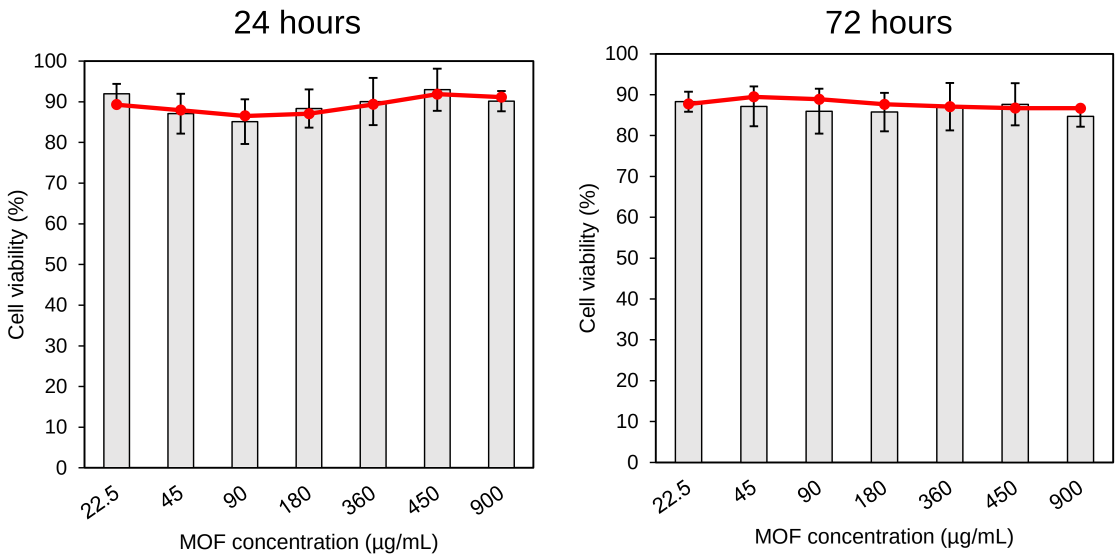

Figure S28. Viability of HeLa cells after 24 h (left) and 72 h (right) incubation with different concentrations of MIP-210(Fe) unloaded (bars) and loaded with NO (red line). The viability of the different conditions was calculated by comparison to a 100% control (only cells). Values expressed as mean ± SD from three independent experiments, each performed with eight replicates.

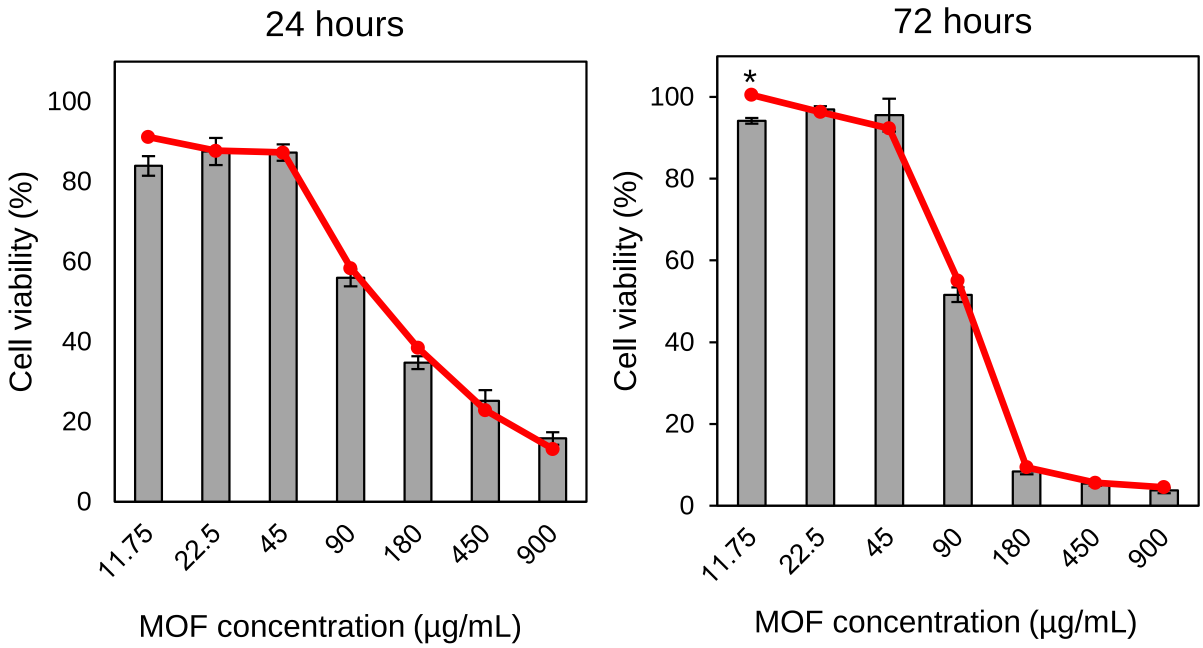

Figure S29. Cell viability of HUVEC cells after 24 h (left) and 72 h (right) incubation with different concentrations of MIP-210(Fe) unloaded (bars) and loaded with NO (red line). The viability of the different conditions was calculated by comparison to a 100% control (only cells). The estimated LD_{50} values of MIP-210(Fe) in HUVEC cells are 144 and 110 mg/L for 24 and 72 hours, respectively. Values expressed as mean ± SD from three independent experiments, each performed with eight replicates. (*P<0.05; NO-loaded MOF *vs*. NO-free MOF)

MIP-210(Fe) toxicity was performed in a range of concentrations (from 11.75 to 900 µg.mL^{-1}) using two distinct cell lines: HeLa and HUVECs (Fig. S28 and Fig. S29). In addition, MIP-210(Fe) loaded with NO was also tested and the effect of NO released in the cell's viability was evaluated.

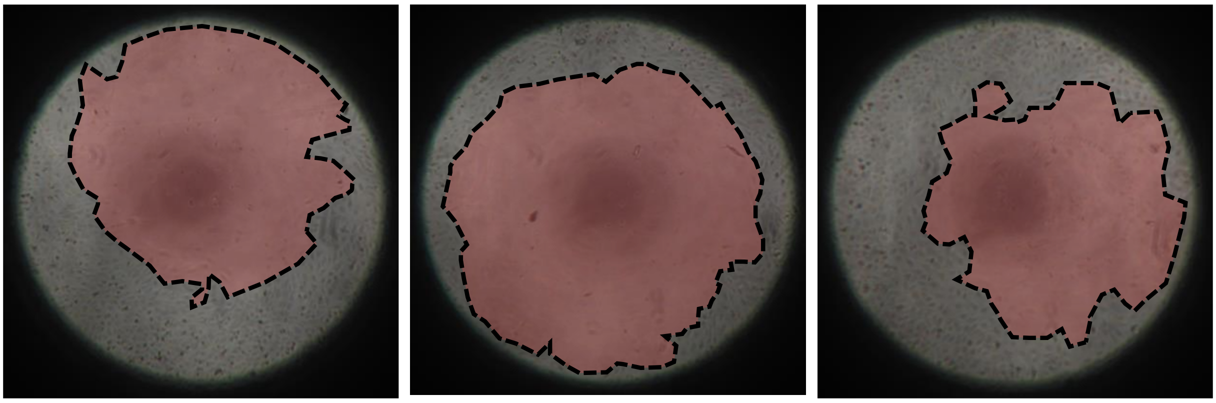

Figure S30. Images of the Oris^{TM} cell migration assay corresponding to the 24 h migration from the representative wells shown in Figure 5A. The figure on the left shows the migrated cells from the control (only cells), figure in the middle represent the cells in the presence of the unloaded MIP-210 and the right figure shows the migrated cells in the presence of the NO-loaded MIP-210. Colored areas were made to evidence the migration zone that is still not occupied by cells.

4.1 Detailed discussion on MIP-210(Fe) biocompatibility and *in vitro* studies

The cytotoxicity was assessed with three different cell lines, HeLa, HEKn and HUVEC, cultivated in RPMI-1640, EpiLife and Medium 200PRF, respectively (Fig. S27-S29). The selection of this cell lines relies on the fact that HeLa cells are one of the most common immortalized cells used for biomaterials toxicity screening, HEKn are a primary cell line representative of the human skin since this NO-releasing MOF is of great interest in wound healing and tissue regeneration applications and HUVEC cells are primary endothelial cells also involved in remodeling processes where NO has an important role (e.g. angiogenesis), which some are explored in this work. In a first study, cells were put in contact with MIP-210(Fe) for 72 and 24 hours at the same concentration used to access the stability (450 μg mL^{-1}; Fig S27). Only a residual toxicity with HeLa cells was observed while *ca.* 30% of primary HEKn cells were not viable anymore. In the case of HUVEC cells, a higher toxicity was observed (only 10% of viable cells after 72 hours) at this high concentration. However, since most NO therapeutic effects reveal efficacy between the pmol to nmol range, the concentration of material is expected to be much lower than 450 μg mL^{-1} to guarantee the release of therapeutic levels of NO, which will also ensure the biocompatibility of the solid. Indeed, Figure S29 evidences that concentrations of MIP-210(Fe) lower than 45 μg mL^{-1} are non-toxic to HUVEC cells (for HeLa cells no significant toxicity was found even at 900 μg mL^{-1}; Fig S28). Thus, MIP-210(Fe) presents a remarkable biocompatibility compared with most of the MOFs studied for the same application, which are often compromised from the beginning by using toxic metals in its framework (e.g. Ni, Co) or by being unstable in any aqueous solution (e.g. HKUST-1). The reason for this high viability might be due to a combination of several factors including the non-toxic material chemistry, lack of degradation products and due to a possible nonexistence of cellular uptake.

The first evidence of the therapeutic potential of this new NO donor to regulate biological functions was assessed *in vitro* by performing endothelial migration and tube formation assays. The cell line selected for these assays was the HUVEC (primary human umbilical vein cells) as the development of new blood vessels is mediated by endothelial cells, which migrate and proliferate in response to growth factors, *e.g.*, vascular endothelial growth factor (VEGF) to form a functional capillary network[21]. NO is known to significantly contribute to the VEGF mediation and its presence was demonstrated to contribute to an enhanced proliferation and angiogenic response in a concentration-dependent manner[22].

An improved migration behavior will allow the endothelial cells to migrate faster throughout the wound gap, and a richer capillary formation will guarantee the uniform distribution of nutrients, growth factors and functional cells, essential to support the recovery of the full wound[23].

To evaluate the NO-loaded MIP-210(Fe) concentration that triggers the positive biological response, we used the viability assay to compare the effects of unloaded and NO-loaded MIP-210(Fe) at different concentrations. Indeed, the NO released from MIP-210(Fe) should be delivered at low concentrations (nM) to exert an angiogenic effect[24,25]. As discussed above, HUVEC viability is affected with MOF concentrations higher than 45 $\mu g.mL^{-1}$. However, at lower MOF concentrations such as 11.75 $\mu g.mL^{-1}$, an enhanced cell viability was observed in presence of NO-loaded MIP-210(Fe) particles compared to cells treated with the NO-free MOF (Fig. S29), which suggests a slight proliferative effect mediated by the released NO. Besides being primary cells, HUVECs are much more sensitive to noxious stimuli than immortalized cells but present a closer behavior to the real systems. For example, HeLa cells show significant resistance to high concentrations of potential toxins[26] and the NO released by MIP-210(Fe) at 900 $\mu g.mL^{-1}$ did not exert any toxic effect on them (Fig. S28).

Bibliography

[1] N. A. Caplan, C. I. Pogson, D. J. Hayes, G. M. Blackburn, *J. Chem. Soc.{,} Perkin Trans. 1* **2000**, 421–437.

[2] M. Feelisch, D. Kubitzek, J. Werringloer, in *Methods Nitric Oxide Res.* (Eds.: M. Feelisch, J.S. Stamler), John Wiley & Sons, Ltd, New York, **1996**, pp. 455–478.

[3] P. E. Blöchl, *Phys. Rev. B. Condens. Matter* **1994**, *50*, 17953–17979.

[4] G. Kresse, J. Furthmüller, *Phys. Rev. B* **1996**, *54*, 11169–11186.

[5] G. Henkelman, B. P. Uberuaga, H. Jónsson, *J. Chem. Phys.* **2000**, *113*, 9901–9904.

[6] V. Georget, V. Baecker, "ImageJ-macros: Wound Healing Tool," **n.d.**

[7] P. S. Wheatley, A. C. McKinlay, R. E. Morris, *Stud. Surf. Sci. Catal.* **2008**, *174*, 441–446.

[8] R. E. Morris, P. S. Wheatley, *Angew. Chemie Int. Ed.* **2008**, *47*, 4966–4981.

[9] N. J. Hinks, A. C. McKinlay, B. Xiao, P. S. Wheatley, R. E. Morris, *Microporous Mesoporous Mater.* **2010**, *129*, 330–334.

[10] B. Xiao, P. S. Wheatley, X. Zhao, A. J. Fletcher, S. Fox, A. G. Rossi, I. L. Megson, S. Bordiga, L. Regli, K. M. Thomas, R. E. Morris, *J. Am. Chem. Soc.* **2007**, *129*, 1203–1209.

[11] J. F. Eubank, P. S. Wheatley, G. Lebars, A. C. McKinlay, H. Leclerc, P. Horcajada, M. Daturi, A. Vimont, R. E. Morris, C. Serre, *APL Mater.* **2014**, *2*, 124112.

[12] L. Ai, L. Li, C. Zhang, J. Fu, J. Jiang, *Chem. – A Eur. J.* **2013**, *19*, 15105–15108.

[13] D. Cattaneo, S. J. Warrender, M. J. Duncan, C. J. Kelsall, M. K. Doherty, P. D. Whitfield, I. L. Megson, R. E. Morris, *RSC Adv.* **2016**, *6*, 14059–14067.

[14] R. V Pinto, S. Wang, S. R. Tavares, J. Pires, F. Antunes, A. Vimont, G. Clet, M. Daturi, G. Maurin, C. Serre, M. L. Pinto, *Angew. Chemie Int. Ed.* **2020**, *59*, 5135–5143.

[15] J. Perdew, K. Burke, M. Ernzerhof, *Phys. Rev. Lett.* **1996**, *77*, 3865–3868.

[16] G. Kresse, D. Joubert, *Phys. Rev. B* **1999**, *59*, 1758–1775.

[17] G. Kresse, J. Furthmüller, *Comput. Mater. Sci.* **1996**, *6*, 15–50.

[18] S. Grimme, J. Antony, S. Ehrlich, H. Krieg, *J. Chem. Phys.* **2010**, *132*, 154104.

[19] L. Wang, T. Maxisch, G. Ceder, *Phys. Rev. B* **2006**, *73*, 195107.

[20] "Vasp TST tools," can be found under http://theory.cm.utexas.edu/vtsttools/, **n.d.**

[21] G. Neufeld, T. Cohen, S. Gengrinovitch, Z. Poltorak, *FASEB J. Off. Publ. Fed. Am. Soc. Exp. Biol.* **1999**, *13*, 9–22.

[22] L. Morbidelli, S. D. and M. Ziche, *Curr. Pharm. Des.* **2003**, *9*, 521–530.

[23] R. Zhan, S. Yang, W. He, F. Wang, J. Tan, J. Zhou, S. Yang, Z. Yao, J. Wu, G. Luo, *PLoS One* **2015**, *10*, e0121551.

[24] P. Zhang, Y. Li, Y. Tang, H. Shen, J. Li, Z. Yi, Q. Ke, H. Xu, *ACS Appl. Mater. Interfaces* **2020**, *12*, 18319–18331.

[25] T. Yang, A. N. Zelikin, R. Chandrawati, *Adv. Sci.* **2018**, *5*, 1701043.

[26] M. J. Duncan, P. S. Wheatley, E. M. Coghill, S. M. Vornholt, S. J. Warrender, I. L. Megson, R. E. Morris, *Mater. Adv.* **2020**, *1*, 2509–2519.
